# Porous polycarbene-bearing membrane actuator for ultrasensitive weak-acid detection and real-time chemical reaction monitoring

**DOI:** 10.1038/s41467-018-03938-x

**Published:** 2018-04-30

**Authors:** Jian-Ke Sun, Weiyi Zhang, Ryan Guterman, Hui-Juan Lin, Jiayin Yuan

**Affiliations:** 1 Department of Colloid Chemistry, Max Planck Institute of Colloids and Interfaces, Potsdam, D-14424 Germany; 2 Department of Chemistry and Biomolecular Science, and Center for Advanced Materials Processing, Clarkson University, 8 Clarkson Avenue, Potsdam, NY 13699 USA; 3 Department of Materials and Environmental Chemistry, Stockholm University, Svante Arrheniusväg 16C, Stockholm, 10691 Sweden

## Abstract

Soft actuators with integration of ultrasensitivity and capability of simultaneous interaction with multiple stimuli through an entire event ask for a high level of structure complexity, adaptability, and/or multi-responsiveness, which is a great challenge. Here, we develop a porous polycarbene-bearing membrane actuator built up from ionic complexation between a poly(ionic liquid) and trimesic acid (TA). The actuator features two concurrent structure gradients, i.e., an electrostatic complexation (EC) degree and a density distribution of a carbene-NH_3_ adduct (CNA) along the membrane cross-section. The membrane actuator performs the highest sensitivity among the state-of-the-art soft proton actuators toward acetic acid at 10^−6^ mol L^−1^ (M) level in aqueous media. Through competing actuation of the two gradients, it is capable of monitoring an entire process of proton-involved chemical reactions that comprise multiple stimuli and operational steps. The present achievement constitutes a significant step toward real-life application of soft actuators in chemical sensing and reaction technology.

## Abstract (web summary)

The design of soft actuators which show high sensitivity and allow for simultaneous interaction with multiple stimuli still remains a challenge. Here the authors demonstrate a highly sensitive proton actuator which allows monitoring of an entire process of chemical reactions that comprise multiple stimuli and operational steps.

## Introduction

Smart soft actuators are capable of mechanical reconfiguration by translating the energy contained within a chemical or physical stimulus into micro-/macroscopic change of their shape and/or size^1–5^. Current actuator devices that respond to a subtle fluctuation in environmental conditions are particularly promising for the development of more sustainable, low-power-consumption actuators and electronic systems such as highly sensitive detectors and diagnostic instruments^6–11^. Among a variety of actuation mechanisms, the solvent-stimulus represents an important one, in which the solvent diffusion into a polymer entity results in heterogeneous volume change in its body, giving rise to micro-/macroscopic shape deformation and/or adaptive movements^12–16^. In some cases, solvents may not trigger actuation directly but serve purely as fluidic carrier to transport the real stimuli, e.g., protons, to reach the site of action^17–22^. In nature, biological proton actuators such as adenosine triphosphate (ATP)-driven proton pumps on the plasma membrane expel protons to create a negative transmembrane potential and an external acidic pH environment that drives the cell expansion^23,24^. These biological actuators have inspired materials scientists to develop proton-active artificial systems including responsive gels, membranes, and shape memory polymers^17–22^. Similar to most other polymer actuators, they suffer from low sensitivity. Usually a high concentration of protons in aqueous solution (≥10^−3^ mol L^−1^) was required in order to produce visual shape deformation or displacement. The realization of sensitive motion at a low proton concentration is challenging and desirable for real-time monitoring of proton-related chemicals. This is particularly the case with weak acids, as they are crucial components in food and chemical industries, in agriculture, and in medicine^25,26^. However, how such acidity is sensed and translated into an appropriate behavioral response (e.g., actuation or electric pulse) is poorly developed. It is especially a dilemma for actuator-based proton sensing mechanism for low limit-of-detection due to inefficient and variable ionization of weak acids in aqueous media in comparison to strong acids at the same concentration. Overall, how to build smart, ultrasensitive actuators for weak acids preferentially in aqueous media is eagerly pursued.

Poly(ionic liquid)s (PILs) as the polymerization products of ionic liquids have attracted growing interest across polymer and materials science owing to their broad physical property window, unprecedentedly rich choices in monomer unit structure, and multifunctional nature for materials design^27–30^. Currently, the advance in PIL chemistry, physics, and engineering is under acceleration, which makes a great impact on a multiple of subfields of chemistry and materials science^31–38^. For example, adaptive solubility of PILs is a striking feature enabling exotic membrane structures that were previously unattainable by other polymers^39,40^. As reported previously^39^, the diffusion of aqueous ammonia solution into a PIL/poly(acrylic acid) blend film produced a nanoporous membrane carrying an unusual electrostatic complexation (EC) gradient along its cross-section, which rendered the membrane an actuation function in contact with solvents. Very recently, the chemistry of poly(1,2,4-triazolium) PILs has emerged and served as polycarbene precursor under a weak base condition, a chemical property that was immediately adopted to immobilize metal clusters of high catalytic activity^41,42^. Overall, these activities in PIL research have been catalyzing new solutions and applications in chemistry and materials design.

In this contribution, by merging the newly discovered PIL-derived polycarbene chemistry with the latest progress in porous polymer fabrication technique, we report here the synthesis of a free-standing nanoporous polycarbene-bearing membrane that serves as an actuator sensor to detect acetic acid (CH_3_COOH) down to ~3.7 × 10^−6^ mol L^−1^ (M) in aqueous media, and could in situ monitor an entire proton-involved chemical reaction event, indicative of their futuristic real-life application in reaction technology. Their advanced functionality stems from two structural gradients engineered simultaneously into a membrane that is capable of independently responding to multiple external stimuli in an intricate, dynamic environment, e.g., a reaction process. We also highlight that the radically enhanced structural complexity and functionality in these systems do not add extra synthetic burden in comparison to the established state-of-the-art porous membrane fabrication process^39,40^.

## Results

### Preparation and characterization of porous membranes

**Fig. 1:**
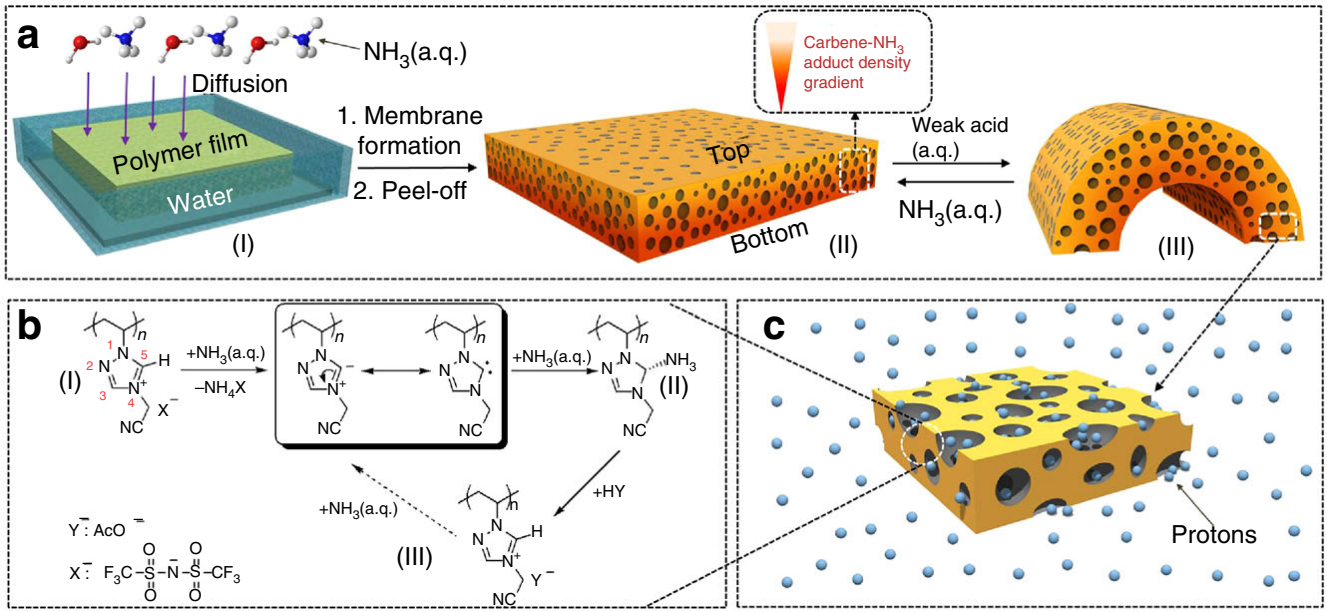
Schematic diagram of the design concept and the proposed chemical structure evolution of the polycarbene-bearing nanoporous Patriaz-TA membrane actuator. **a** A physical blend film of a Patriaz-TA mixture after aqueous NH_3_ treatment, leads to electrostatic complexation (EC) between the 1,2,4-triazolium cations on Ptriaz and the carboxylate anions on TA; Note that a part of 1,2,4-triazolium cations were deprotonated by reacted with NH_3_ to form carbene-NH_3_ adduct (CNA) unit. A decreasing density in CNA was formed, from the membrane top to the bottom. The membrane can respond to external weak acid with bottom part inwards and restore its initial 1,2,4-triazolium state in the presence of weak base (a.q. NH_3_). **b** The chemistry of reversible formation-decomposition of CNA in poly(1,2,4-triazolium) chains guides membrane actuator for ultrasensitive proton sensing in aqueous solution in **c**

The chemical synthesis started with a PIL poly[4-cyanomethyl-1-vinyl-1,2,4-triazolium bis(trifluoromethane sulfonyl)imide] (termed “Ptriaz,” chemical structure shown in Supplementary Methods), which carries a cyanomethyl substituent along its poly(1,2,4-triazolium) backbone. Its chemical structure and apparent molecular weight were characterized and confirmed by proton nuclear magnetic resonance (^1^H NMR) spectroscopy and gel permeation chromatography (Supplementary Fig. 1–3). The porous membrane was prepared according to a modified protocol of our previous method^40^. Briefly, a mixture solution of Ptriaz and trimesic acid (TA) at a 1:1 equivalent molar ratio of triazolium/COOH in *N,N*-dimethylformamide (DMF) was drop-cast onto a glass plate and then dried at 80 °C for 2 h into a thin sticky film, before it was immersed in an aqueous ammonia solution (7 × 10^−2^ M) for 2 h. A free-standing membrane (termed “Ptriaz-TA membrane”) was easily peeled off (Fig. 1) (Supplementary Fig. 6) under such optimized condition (Supplementary Fig. 7–9). For the sake of clarity, the membrane surfaces facing the glass plate and the ammonia solution were denoted as BOTTOM and TOP surface, respectively. The membrane formation mechanism can be referred to previous publications^39,40^. The NH_3(a.q.)_ is key here for membrane formation, as the diffusion of ammonia molecules into the supported Ptriaz/TA blend film neutralizes and triggers EC from top to bottom; meanwhile water molecules that have diffused into the film introduce phase separation^39,40^. The as-produced membrane featured a hierarchically porous morphology with pore size ranging from 50 nm to 2 µm (Supplementary Fig. 8) and a density gradient in EC degree along its cross-section decreasing from the membrane top to bottom (Supplementary Fig. 10–11). These are two intrinsic characteristics for membranes fabricated by this method^39,40^. Despite the high porosity, its tensile strength and strain at break are up to 0.66 MPa and 4%, respectively (Supplementary Fig. 12), which are comparable to those of tissue papers^43^.

In the 1,2,4-triazolium cation ring the C5 proton is known to be active and undergoes easier deprotonation than other N-heterocyclic cations, such as treatment by weak base, e.g., NaBH_4_^41^. Inspired by this chemistry, a similar reaction of a triazolium IL with NH_3_ in *d*_*6*_-DMSO was conducted. In the ^1^H NMR spectrum the disappearance of the C5 proton resonance after addition of NH_3_ was observed, proving the occurrence of NH_3_-induced deprotonation reaction (Supplementary Fig. 13). When NH_3_ is added in excess, it reacts with the active N-heterocyclic carbene in situ to form a carbene-NH_3_ adduct (CNA). A new resonance at 160 ppm in the ^13^C NMR spectrum was observed here and is consistent with the formation of a CNA (Supplementary Fig. 14)^44–46^. More interestingly, the CNA can be reverted to the 1,2,4-triazolium cation via treatment with a weak acid, such as acetic acid (Supplementary Figs. 13–14). Therefore, the 1,2,4-triazolium cation and CNA can be chemically cycled in a mild fashion via weak acid/base treatment, rather than the HCl/1,8-diazabicyclo[5.4.0]undec-7-ene (DBU) pair reported previously^47^. Since the porous membrane is abundant with 1,2,4-triazolium cations as the major component in the polymer, the same chemistry controlled by NH_3_ occurs to the membrane. Similar to the EC degree gradient that is produced by a NH_3_ diffusion process, here a density gradient of CNA units along the membrane cross-section is present.

The asymmetric CNA distribution was supported first by surface zeta potential measurements on both membrane surfaces. The top one shows a zeta potential value of +18.9 mV, which is lower than the bottom (+29.7 mV). This result is not surprising because the NH_3_ concentration is the highest on the top surface, thus greater amounts of deprotonation/neutralization occurred (Supplementary Fig. 15), reducing the number of charges.

**Fig. 2:**
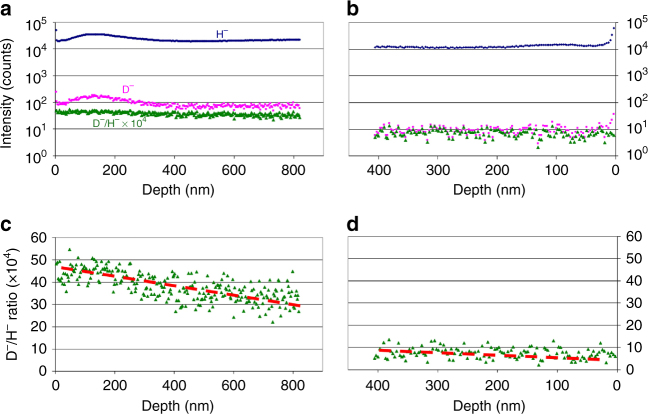
The ToF-SIMS results of Ptriaz-TA membrane. Depth profiles of H¯ and D¯ for the membrane top (**a**) and bottom surface (**b**). The D¯/H¯ ratio in **a**, **b** is multiplied by 10^4^, shown in **c**, **d** are D¯/H¯ ratios plotted in a linear scale for the depth profiles closed to the top and bottom surfaces of membrane, respectively. The local gradient distribution of deuterium was also revealed in **c** and **d**. The gradual decrease of the deuterium amount as increasing the depth from top surface was observed (indicated by a dashed red line). Note: the natural abundance of deuterium corresponds to a D¯/H¯ ratio of 1.6 × 10^–4^

Further evidence of a CNA density gradient was given by time-of-flight secondary ion mass spectrometry (ToF-SIMS) measurements. Prior to the measurement, the Ptriaz-TA membrane was treated by a CH_3_COOD solution in D_2_O, which as discussed previously transformed CNA to 1,2,4-triazolium with deuterium at the C5 position. This treatment in fact replaces the CNA density gradient by a deuterium one across the membrane. It should be noted that the deprotonated TA itself is unlikely to accept deuterium from CH_3_COOD because of the *pK*_a_ series: *pK*_a_ (Ptriaz, 8.62) < *pK*_a_ (CH_3_COOH, 4.76) < *pK*_a_ (TA, 2.98). This rule was consistent with the observation in ^1^H NMR spectroscopy measurements in *d*_6_-DMSO, in which deprotonated TA after treatment with CH_3_COOH showed a negligible signal at 13.5 ppm belonging to the acidic protons in TA (Supplementary Fig. 16). It was further supported by FT-IR spectra that little-to-no change was identified for the –COO^−^ vibration band at 1615 cm^−1^ between the original and acetic acid-treated membrane (Supplementary Fig. 17). These experiments demonstrate that the incorporated deuterium was majorly if not exclusively captured by the C5 carbon to restore the triazolium cations. Compared with the natural abundance of deuterium corresponding to a fixed D¯/H¯ ratio of 1.6 × 10^−4^, the ToF-SIMS results unequivocally confirmed that the deuterium concentration levels close to the top part were more than five times higher than the bottom (averagely 38.2 vs. 7.5 in Fig. 2). The local gradient distribution of deuterium on the top and at the bottom region was also observed to decrease gradually from the top to the bottom, in good agreement with the CNA density gradient.

### Proton-responsive actuation performance of membranes

**Fig. 3:**
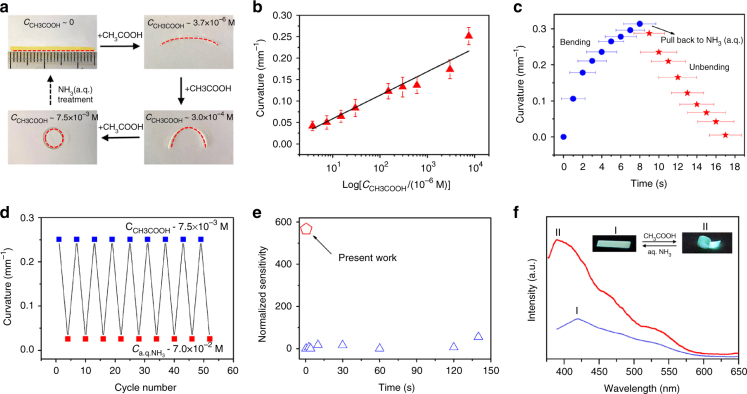
The actuation of the Ptriaz-TA membrane actuator. **a** Shape deformation of a Ptriaz-TA membrane (1 mm × 25 mm × 50 µm) in response to the addition of CH_3_COOH in water at 25 °C. Note: the membrane on the up-left was a top view and the rest was side view. The red dashed line indicates the membrane bottom. **b** The linear correlation of the membrane curvature against CH_3_COOH concentration (The method for statistical analysis is based on “arithmetic mean,” which is also used to other data analyses in this work). **c** Plot of curvature against time for the membrane actuator in CH_3_COOH-water solution (7.5 × 10^−3^ M) and back in aq. NH_3_ (7 × 10^−2^ M). **d** The recyclability test upon alternatively treating the membrane with a.q. NH_3_ and a.q. CH_3_COOH solution. **e** Plot of “normalized sensitivity” against time for the membrane actuator in current work (red pentagon) and from literature results (blue triangles) (Supplementary Table 1). **f** Fluorescence photographs (taken under *λ* = 365 nm) and spectra (*λ*_ex_ = 365 nm) record from the two states of membrane (1 mm × 25 mm × 50 µm). I a.q. NH_3_ treated Ptriaz-TA membrane; II after soaking in a.q. CH_3_COOH solution

The CNA density gradient, as a previously unexplored structural asymmetry in a membrane, enables the Ptriaz-TA membrane as proton actuator. Here CH_3_COOH (*pK*_a_ = 4.76) as one of the most common weak acids was chosen as a model compound. Initially, a flat membrane (1 mm × 25 mm × 50 µm) was placed in water (Fig. 3a, top left). Its bottom steadily bent inwards (curvature ~ 0.042 mm^−1^, calculated according to Supplementary Method) in an aqueous CH_3_COOH solution at merely *C*_CH3COOH_ - 3.7 × 10^−6^ M (Supplementary Fig. 19 shows the concentration dependent shape deformation of the membrane). The membrane arch gradually advanced into a closed loop at *C*_CH3COOH_ - 7.5 × 10^−3^ M (Fig. 3a, bottom left). The membrane can further curl at a higher CH_3_COOH concentration up to 1.25 × 10^−2^ M, giving an estimated dynamic actuation range of 3 × 10^3^ (Supplementary Fig. 20). Figure 2b exhibited the curvature vs. log *C*_CH3COOH_ plot, which satisfies a linear fit, indicating a decisive role of proton in the actuation mechanism that will be discussed later. Upon immersion in a 7 × 10^−2^ M aqueous NH_3_ solution the membrane unbent fully to its flat state. The kinetic of its reversible actuation was investigated (Fig. 3c), reaching a circle within 8 s in contact with a 7.5 × 10^−3^ M a.q. CH_3_COOH solution (Supplementary Movie 1), and 9 s for recovery (Supplementary Movie 2). This bending–unbending cycle can be repeated at least 50 times without mechanical fatigue (Fig. 3d). The maximum bending force was detected to be 32 mg, i.e., ~ 320 mN, which is 16 times of the actuator’s weight (2 mg). In a control experiment without the membrane actuator, only negligible forces can be observed (<1 mg, see details in Supplementary Fig. 21).

The sensitivity performance of the Ptriaz-TA membrane was compared with those reported in literatures. It is known that bending actuation is inversely proportional to membrane thickness (Supplementary Fig. 22), thus both “normalized sensitivity” (curvature × thickness) and the apparent sensitivity were plotted to compare our membrane with previous proton actuators (Supplementary Table 1). It is found that the Ptriaz-TA membrane actuator is at least one order of magnitude more sensitive (Fig. 3e, Supplementary Table 1). Moreover, the actuation rate is important for a fast feedback. The normalized sensitivity was plotted against time, and the best performance actuators are found in the top left of this diagram. It is seen that our membrane actuator outperforms conventional proton responsive polymer actuators—allowing for the simultaneous combination of ultrafast and ultrasensitive actuation that is rarely observed.

## Discussion

The underlying actuation mechanism toward weak acids was investigated in detail. The asymmetric swelling and the resulting volume expansion across the membrane is commonly the source for actuation. When immersed in an a.q. CH_3_COOH solution, the neutral CNA sites in the Ptriaz-TA membrane are protonated into 1,2,4-triazolium cations. Since the CNA density decreases from the top to bottom along the cross-section of the membrane, more 1,2,4-triazolium units will be freshly generated on the membrane top than the bottom. It is known that equilibrium swelling of cross-linked polyelectrolytes is dependent on local charge density ^22^, and the enhanced ionic density on the polymer chain induces inter-/intramolecular expansion due to mutual electrostatic repulsion among neighboring chains. Thus, the increase in ion concentration through the weak acid treatment is more pronounced on the membrane top than bottom, leading to stronger swelling and larger volume expansion on the top to direct bending, with the bottom part inward. This deduction is supported by a control experiment, in which the membrane with random CNA distribution failed to actuate under the same conditions (Supplementary Fig. 23). As mentioned previously, the acidity follows the sequence of *pK*_a_ (Ptriaz, 8.62) < *pK*_a_ (CH_3_COOH, 4.76) < *pK*_a_ (TA, 2.98), thus CH_3_COOH will protonate the CNA unit rather than the deprotonated TA. As such, CNA as a relatively weaker acid containing highly active sites (C-5 in the heterocyclic ring) accounts for the protonation and is the key structural motif for responding to acids. It was also noted that the present membrane actuator is nanoporous (50 nm to 2 µm in pore size), in stark contrast to common dense membrane actuators. The pore channels accelerate proton transport into the membrane, which circumvent the need for a high proton concentration normally built up to drive molecular diffusion and penetration into dense matters, thus leading to a higher sensitivity toward weak acids. A control experiment using nonporous membrane showed a rather slower bending rate when tested at *C*_CH3COOH_ - 7.5 × 10^−3^ M (Supplementary Fig. 24). Given the same chemical nature of these membranes, unambiguously the pores are critical to improve their actuation speed.

To emphasize the unique role of the 1,2,4-triazolium-derived unit in membrane actuation, an ionically complexed membrane stripe in the same size (1 mm × 25 mm × 50 µm) prepared from an imidazolium-based PIL (termed “PIm,” Supplementary Fig. 4) and TA was used as actuator (termed “PIm-TA membrane”) toward CH_3_COOH under the same condition. The PIm-TA membrane bent weakly at *C*_CH3COOH_ - 3.7 × 10^−6^ M (Supplementary Fig. 25b) (curvature: 0.019 mm^−1^) in comparison with that of Ptriaz-TA (curvature: 0.042 mm^−1^). In fact, the linear PIm in an a.q. NH_3_ solution can be deprotonated only partially up to 30% to form CNA, as evidenced in the ^1^H NMR spectra in *d*_6_-DMSO in Supplementary Fig. 26. The kinetic deuterium exchange experiment in D_2_O supported that the active C-2 proton in imidazolium ring is slower to be exchanged in comparison to the C-5 proton in 1,2,4-triazolium ring (^1^H NMR spectrum in Supplementary Fig. 27)^48^. The latter can be totally exchanged within 5 min, whereas in the same period the former reached only 45% H-D exchange. The chemically introduced nitrogen atom at N2 position in the 1,2,4-triazolium ring was previously reported crucial for carbene formation under weak base condition, in good agreement with our observation here^41^. We prepared also a membrane from a pyridinium PIL (Termed “PPy,” Supplementary Fig. 5) and the actuator (Termed “PPy-TA”) (1 mm × 25 mm × 50 µm) did not actuate at all because of no carbene formation in a pyridinium ring (Supplementary Fig. 25c).

Intriguingly, the protonation/deprotonation reaction on 1,2,4-triazolium is accompanied with a change in the electronic structure of the 1,2,4-triazolium ring, leading to switchable fluorescence emission simultaneously to its actuation, as shown in Fig. 3f. When the membrane (1 mm × 25 mm × 50 µm) was curled at *C*_CH3COOH_ - 7.5 × 10^−3^ M, the fluorescence intensity increased and the color switched from weak yellowish blue (*λ*_max_ = 420 nm, shoulder *λ*_sh_ = 465, 540 nm) to bright blue (*λ*_max_ = 389 nm, *λ*_sh_ = 540 nm). The fluorescence emission spectrum of CH_3_COOH-treated Ptriaz-TA membrane was consistent with that of native Ptriaz (Supplementary Figs. 28 and 29), while the influence of TA is negligible (Supplementary Fig. 30), indicating the occurrence of protonation of CNA during CH_3_COOH treatment. This hypothesis was supported by placing membranes at different *C*_CH3COOH_ (Supplementary Fig. 31), where a gradually enhancing emission intensity accompanied with a blue shift of emission plot was observed. This behavior is understandable as more CNA units in the membrane are transferred into 1,2,4-triazolium at a higher *C*_CH3COOH_. Since the fluorescence emission of ionic liquids and PILs is known to stem from interaction of associated energy species, i.e., short and long-range spatial correlations of the cation–anion and cation–cation pairs^49–51^, the CNA-containing membrane enhanced its emission intensity at a higher *C*_CH3COOH_ by regenerating more ion pair species. The yellowish blue fluorescence in the native membrane was restored by immersion of the CH_3_COOH-treated membrane in a.q. NH_3_ solution (7 × 10^−2^ M) (Fig. 3f). The protonation–deprotonation process varies the electronic struture of Ptriaz acompanied with change of emission spectra^52^, thus the reversible fluorescent switch can be expectantly used as a fluorometric sensor together with the actuation (Supplementary Fig. 31).

**Fig. 4:**
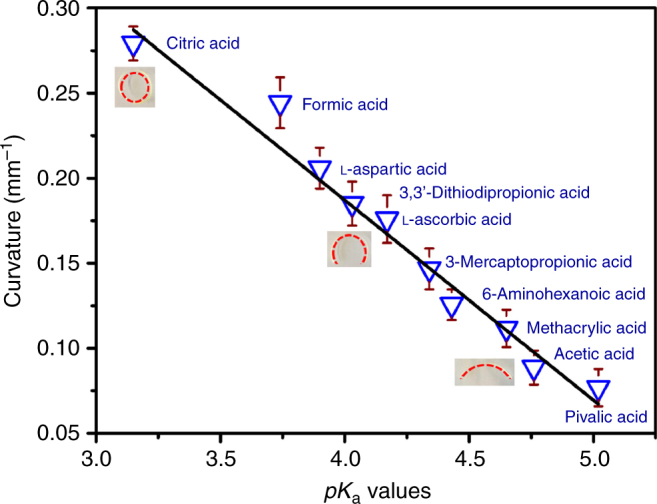
The detection of a broad range of weak-acids with different *pK*_a_ values by Ptriaz-TA membrane actuator. The experiment was conducted by immersing a membrane stripe (1 mm × 25 mm × 80 µm) in a.q. solutions of weak acids at a concentration of 3 × 10^−4^ M. Insets: photographs of the membrane stripe at different acid solutions. The red dashed line in the insets indicates the membrane bottom. The black line is a linear fit of curvature vs. *pK*_a_

The high sensitivity of the Ptriaz-TA membrane actuator allows for functionality unattainable from common examples, such as discriminating specific molecule quality with high accuracy. Since the *pK*_a_ of weak acids determines the degree of ionization in the membrane, we can in principle discriminate between weak acid species by actuation. Lower *pK*_a_ values of weak acids at an identical concentration corresponds to a higher proton concentration thus leading to larger bending curvature. As shown in Fig. 4, the present actuator can sense weak acids from their *pK*_a_ values ranging from 3 to 5 (Supplementary Table 2), which comprises most water-soluble weak acids. A nearly linear relation between curvature and *pK*_a_ is identified, which can be a guidance for weak acid detection.

**Fig. 5:**
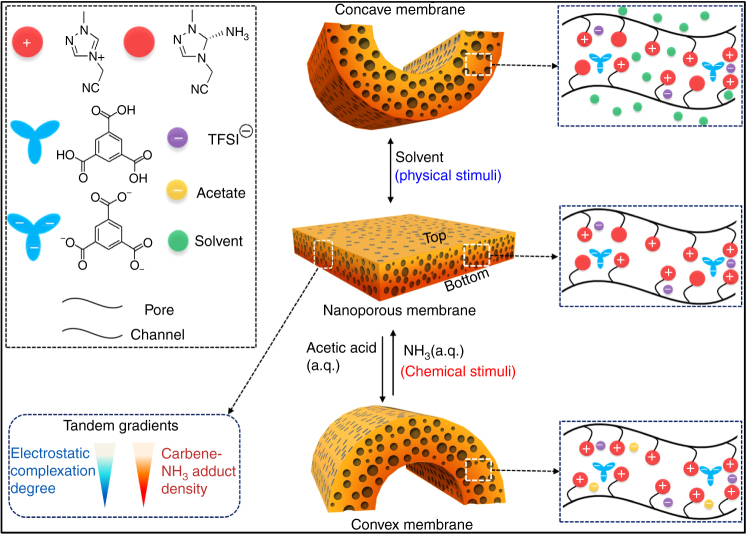
Schematic illustration of bending mechanism derived from the tandem gradients of membrane actuator. Each gradient can independently respond to external chemical/physical stimuli, e.g., the EC degree gradient undergoes actuation once occurrence of solvent–Ptriaz interaction, leading to concave membrane with bottom surface outwards, while the CNA density gradient display actuation once reacting with proton, generating a convex membrane with bottom surface inwards. In the concurrent presence of these two stimuli, two competing bending actuations occur and the dominant one will determine the final curvature

**Fig. 6:**
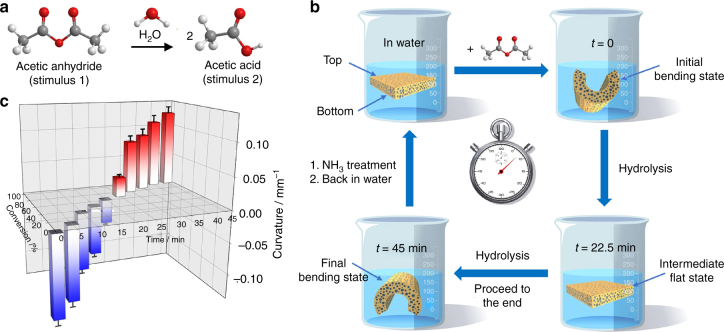
The cascade actuation of tandem-gradient Ptriaz-TA membrane for monitoring a chemical reaction. **a** Ball-stick models represent of two stimuli (the origin reagent acetic anhydride as stimuli 1, and the acetic acid product as stimuli 2) driven for the cascade actuation. **b** Schematic illustration of a cascade motion of a membrane actuator along an entire process of hydrolysis chemical reaction of acetic anhydride in water. **c** 3D histogram by plotting the conversion of acetic anhydride against the corresponding curvature of a membrane stripe (1 mm × 25 mm × 120 µm) in Supplementary Table 3 (the negative value of curvature means the membrane bending with bottom part outwards; positive value with bottom part inwards)

The introduction of a CNA density gradient complicates the membrane structure, which already carries an EC degree gradient that undergoes actuation toward organic solvent stimulus due to solvent–Ptriaz interaction (Supplementary Fig. 32), a mechanism similar to our previous work^39^. Both gradient elements organized in a tandem manner, i.e., the EC degree and the CNA density, decrease from top to bottom, and can independently respond to external stimuli. The difference is, the CNA density gradient bends the membrane with the bottom surface inwards upon acid treatment, while the influence of the EC degree with bottom surface outwards upon exposure to solvent (Fig. 5). In the concurrent presence of these two stimuli, two competing bending actuations occur and the dominant one will determine the final curvature, i.e., the actuation is now a joint rather than individual effect of two bending forces. The competing effect of the two gradient elements opens up a new dimension to control the actuation behavior for unexplored applications^53^, for example, in reaction monitoring technology. Ideally in a chemical reaction, the starting reagent and the product can individually interact with the above-mentioned two gradients to make a dynamic bending of the membrane along the reaction process. A model reaction to demonstrate this prototype reaction-monitor here is the hydrolysis of acetic anhydride in water (Fig. 6a). First, a flat tandem-gradient Ptriaz-TA membrane was placed in aqueous solution. When acetic anhydride, which can dissolve thus strongly interact with Ptriaz (Supplementary Fig. 33), was added at a concentration of 4.5 × 10^−4^ M, it initially bent with its top surface inward due to the interaction between acetic anhydride and Ptriaz (Fig. 6b). This bending signalizes the reaction start (*t* = 0). Along the reaction, one acetic anhydride was gradually transformed into two equivalent CH_3_COOH, which is a trigger molecule to bend the membrane oppositely. As such, the membrane stepwise unbends to a flat intermediate state (*t*_intermediate_ = 22.5 min) and then continuously curved with bottom part inwards till all acetic anhydrides were hydrolyzed, which is defined as the reaction end (*t*_end_ = 45 min). The time-dependent detection of the hydrolysis reaction of acetic anhydride by ^1^H NMR spectra is recorded, and is paired with the development of the membrane curvature (Fig. 6c). It is shown that our model reaction ends up in 45 min with a final curvature at 0.124 mm^−1^ (Fig. 6c). By plotting the conversion of acetic anhydride against the corresponding curvature in Supplementary Table 3, a 3D calibration plane is reached (Supplementary Fig. 34). Assisted by this plot, researchers are able to “read” on-line reaction process by temporally recording the curvature of the membrane in the reaction mixture. It should be said that the Ptriaz-TA membrane has indeed the function to real-time monitor an entire chemical reaction event that combines two stimuli (acetic anhydride and acetic acid) and multiple operational steps, here the addition of the acetic anhydride, the hydrolysis reaction and the post-reaction NH_3_ treatment to restore the flat shape for the next use. Worth to mention is that this monitoring function is not limited to hydrolysis reaction but to reactions where protons are involved.

In conclusion, a tandem-gradient nanoporous membrane actuator was synthesized, which carries two structural gradients individually in an electrostatic complexation degree and in a carbene-NH_3_ adduct density along the membrane cross-section. These two gradients could independently exert response to organic solvents/compounds and weak acids, respectively, and in the presence of these two stimuli their joint effect will determine the true physical motion. In spite of the simplicity of the actuator fabrication process, the membrane reaches a high level of structural complexity and actuation functionality. It expresses the highest sensitivity among all soft proton actuators toward acetic acid at in aqueous media, and it is able to monitor an entire process of chemical reactions that combines multiple steps and carries multiple stimuli with competitive domination. Our design concept here is easy to implement and applies fundamental chemistry in actuator design for real-life application.

## Methods

### Synthesis of polymer materials

The detail of synthesis of PIL polymers can be found in Supplementary Methods

### Preparation of Ptriaz-TA membrane

The membrane actuator was fabricated with procedure as follows: Briefly, a mixture of Ptriaz (100 mg) and TA (16.8 mg) was dissolved in 1.5 mL of dimethylformamide (DMF) solvent. After cast on to a glass plate to form a thin liquid film and dried at 80 °C for 2 h, the sticky thin film attached to the glass plate was immersed into an a.q. ammonia solution (7 × 10^−2^ M, 20 °C) for 2 h. Then, a free-standing membrane was peeled off from the substrate (termed as Ptriaz-TA membrane). The as-prepared membrane exhibited a porous structure characterized by SEM (Supplementary Fig. 7c).

### Preparation of PIm-TA membrane

The synthetic procedure is similar to Ptriaz-TA membrane except by changing of Ptriaz into PIm at equal amount of monomer unit (molar ratio).

### Preparation of PPy-TA membrane

The synthetic procedure is similar to Ptriaz-TA membrane except by changing of Ptriaz into PPy with equal amount of monomer unit (molar ratio).

### Investigation of deuterium distribution along membrane cross-section **via** ToF-SIMS

The membrane was examined using an ION-TOF (GmbH) TOF-SIMS IV equipped with a Bi cluster liquid metal ion source. A 25 keV Bi^3+^ cluster primary ion beam pulsed at 10 kHz was used to bombard the sample surface to generate secondary ions. The positive or negative secondary ions were extracted from the sample surface, mass separated, and detected via a reflectron-type of time-of-flight analyzer, allowing for parallel detection of ion fragments having a mass/charge ratio (*m/z*) up to ~900 within each cycle (100 µs). A pulsed, low energy electron flood was used to neutralize sample charging. This technique is extremely surface-sensitive, probing typically the top 1–3 nm of the sample. The detection limits are in the range of ppb–ppm, depending upon the ion yield of different elements or species. The intensities of a species measured in different samples are useful in estimating its relative changes/abundance in those samples.

Imaging the entire cross-section of the membrane was not successful. Instead, we depth profiled both from the membrane top and bottom surface (i.e., the side contacting the glass substrate). In order to depth profile the film, a 10 keV C_60_^+^ ion beam was used to sputter the surface in an area of 300 µm × 300 µm and negative ion mass spectra were collected at 128 × 128 pixels over an area of 200 µm × 200 µm within the sputtered area. The depth profile data were obtained by sputtering the surface with the C_60_^+^ beam for 5 s followed by a 1 s pause before Bi^3+^ was used to analyze the newly-generated surface (collecting five scans). Depth was calibrated by measuring, using a stylus-type of profilometer, the crater generated on a PMMA film deposited on a Si wafer (Supplementary Fig. 18).

### Chemicals, materials, and characterization

1-Vinyl-1,2,4-triazole (98%), trimesic acid (TA or benzene-1,3,5-tricarboxylic acid, 95%), aqueous ammonia (28 wt%), poly(4-vinylpyridine) (average *M*_w_ ~160,000 g/mol), butylated hydroxytoluene (BHT, 98%, stabilizer), *N,N’*-azobisisobutyronitrile (AIBN, 98%, recrystallized from methanol), acetic acid (glacial, ≥99.85%), and all organic solvents and acids were purchased from Sigma-Aldrich/Acros. 1-Vinylimidazole (99%) and bromoacetonitrile (BrCH_2_CN, 96%) were purchased from Alfa Aesar. Lithium bis(trifluoromethylsulfonyl)imide (LiTFSI, >98%) was obtained from TCI chemicals. All organic solvents and acids were of analytical grade and used as received.

^1^H and ^13^C nuclear magnetic resonance (^1^H-NMR and ^13^C-NMR) spectroscopy measurements were conducted at room temperature on a Bruker Ascend-400/600 spectrometer in different deuterated solvents. Scanning electron microscopy (SEM) was performed on a GEMINI LEO 1550 microscope operating at 3 kV. Samples were sputtered with gold before measurement. Elemental (sulfur) analysis of the membrane cross-section was measured by EDX method (Oxford instruments) via scanning electron microscopy (DSM 940 A, Carl Zeiss AG). The fluorescence spectra were recorded on a LS-50B, Perkin Elmer instrument. Gel permeation chromatography (GPC) was performed using NOVEMA Max linear XL column with a mixture of 80% of aqueous acetate buffer and 20% of methanol. Conditions: flow rate 1.00 mL min^−1^, PS standards (bought from Company PSS) using RI detector-Optilab-DSP-Interferometric Refractometer (Wyatt-Technology). Fourier transform infrared spectroscopy (FT-IR) spectra were recorded on a BioRad 6000 FT-IR spectrometer; samples were measured in solid state using a Single Reflection Diamond ATR. Surface zeta potential was measured on Zetasizer nano ZS. Tensile tests were measured on an HY-0305 materials testing machine (shanghai hengyi instrument Co., Ltd.) at an extension speed of 0.6 mm min^−1^.

### Data availability

The data that support the findings of this study are available from the corresponding author on reasonable request.

## Electronic supplementary material

Supplementary Information

Description of Additional Supplementary Files

Supplementary Movie 1

Supplementary Movie 2
