# Supplementary material for: Porous polycarbene-bearing membrane actuator for ultrasensitive weak-acid detection and real-time chemical reaction monitoring
Source: Nat Commun. 2018 Apr 30;9:1717. doi: 10.1038/s41467-018-03938-x (PMC5928224; doi:10.1038/s41467-018-03938-x)
Supplement: Supplementary file 1 — Supplementary Information [file 41467_2018_3938_MOESM1_ESM.pdf]

Supplementary Information

Porous polycarbene-bearing membrane actuator for ultrasensitive weak-acid detection and real-time chemical reaction monitoring

Sun et al.

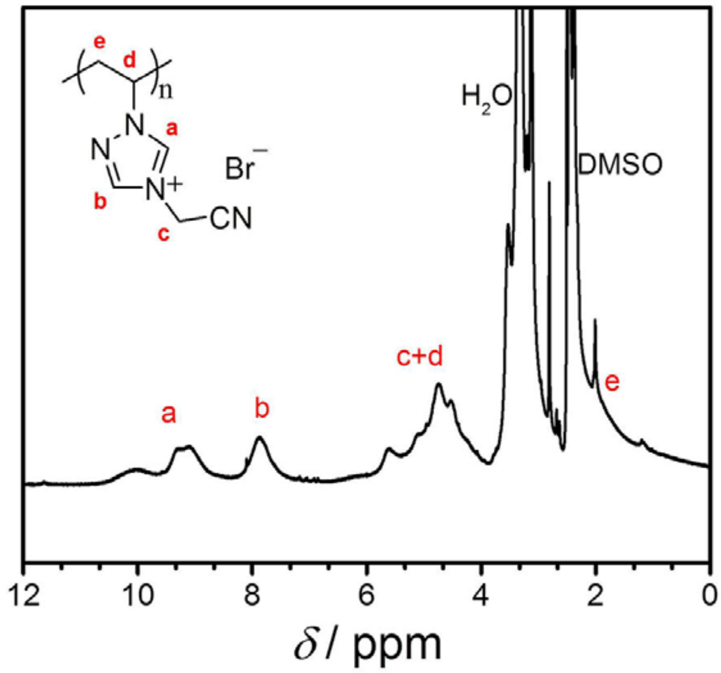

Supplementary Figure 1 | Chemical structure and ^{1}H NMR spectrum of poly(4-cyanomethyl-1-vinyl-1,2,4-triazolium bromide) (PtriazBr).

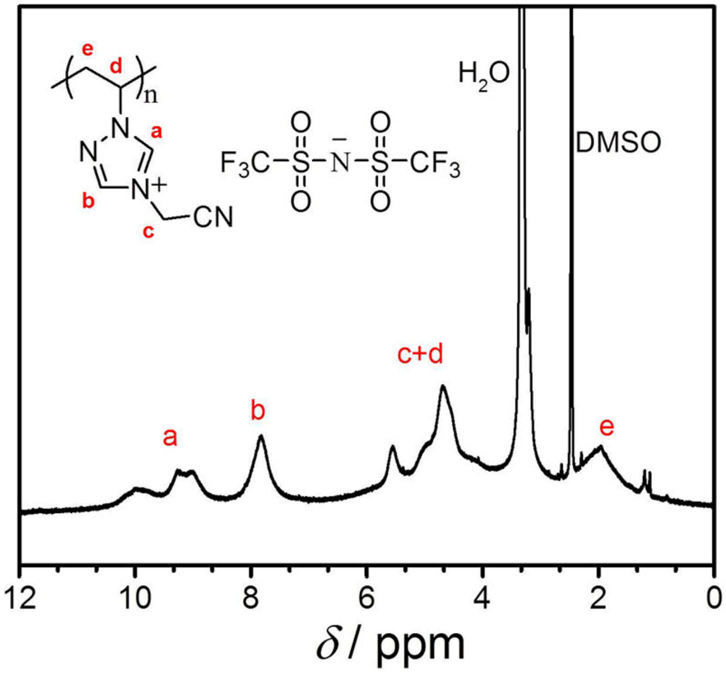

Supplementary Figure 2 | Chemical structure and ^{1}H NMR spectrum of poly(4-cyanomethyl-1-vinyl-1,2,4-triazolium bis(trifluoromethanesulfonyl)imide) (Ptriaz).

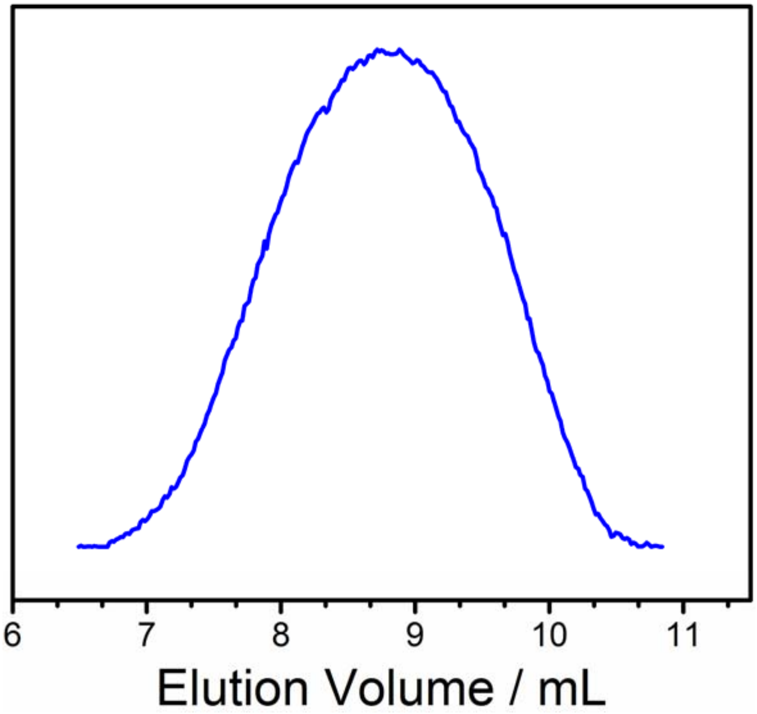

Supplementary Figure 3 | GPC trace measured for poly(4-cyanomethyl-1-vinyl-1,2,4-triazolium bromide) (PtriazBr).

The apparent number-average molecular weight and PDI value of poly(4-cyanomethyl-1-vinyl-1,2,4-triazolium bromide) (PtriazBr) was measured to be 3.40×10^5 g mol^{-1} and 2.70, respectively (measured by GPC, eluent: water with a mixture of 80% acetate buffer and 20% methanol). Poly(4-cyanomethyl-1-vinyl-1,2,4-triazolium bis(trifluoromethanesulfonyl)imide) (Ptriaz) was prepared by anion exchange of PtriazBr with LiTFSI salt. Therefore, the apparent number-average molecular weight of Ptriaz is calculated to be 6.52×10^5 g mol^{-1}.

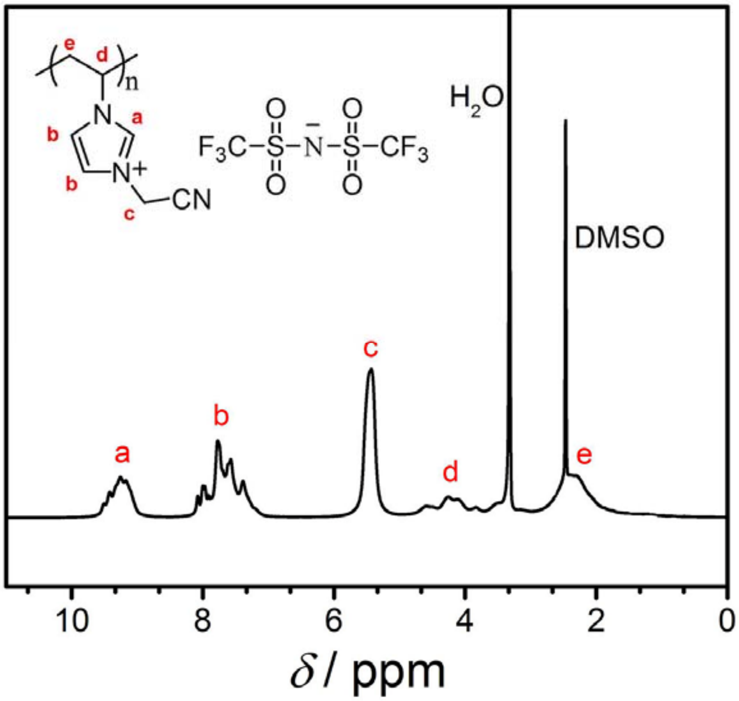

Supplementary Figure 4 | Chemical structure and ^{1}H NMR spectrum of poly(3-cyanomethyl-1-vinylimidazolium bis(trifluoromethanesulfonyl)imide) (PIm).

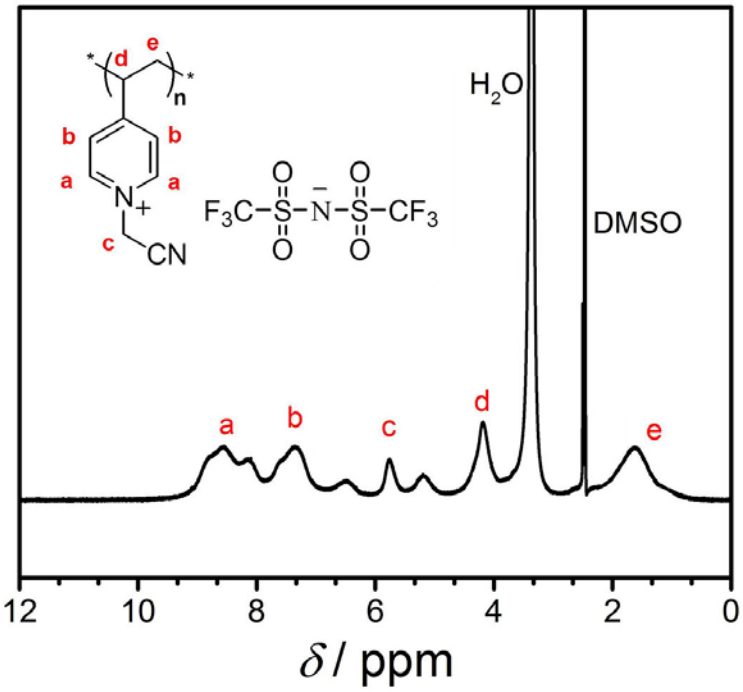

Supplementary Figure 5 | Chemical structure and ^{1}H NMR spectrum of poly(1-cyanomethyl-4-vinyl-pyridinium bis(trifluoromethanesulfonyl)imide) (PPy).

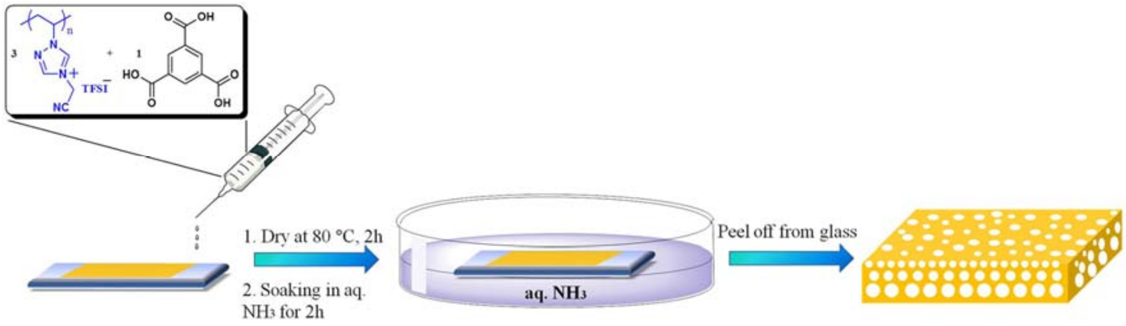

Supplementary Figure 6 | Schematic illustration of preparing Ptriaz-TA membrane.

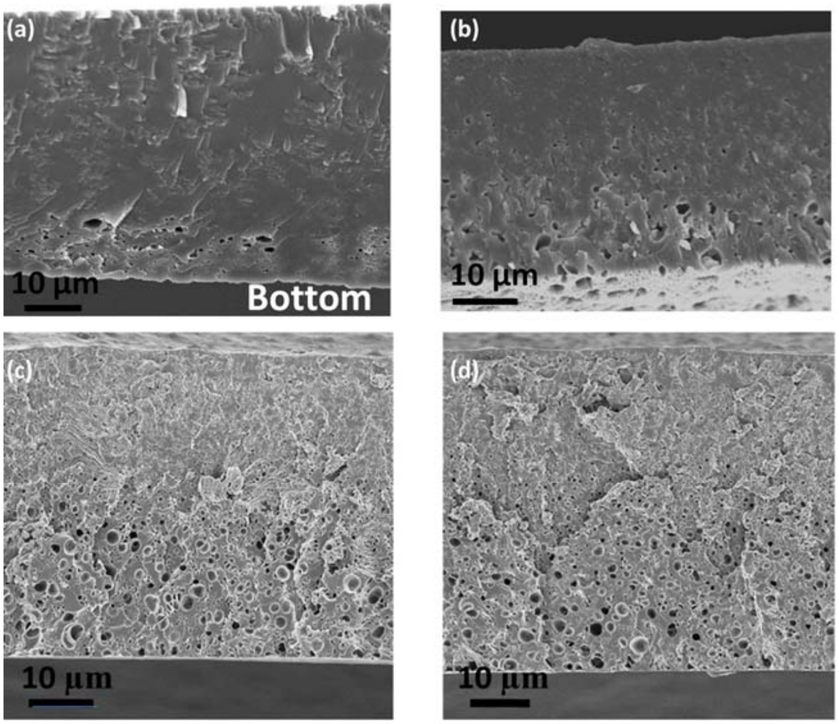

Supplementary Figure 7 | Cross-section SEM images of membranes (The bottom surface is labeled in a which applies also to b-d). (a) The Ptriaz-TA blend film after soaking in an *a.q.* ammonia solution for 10 min, (b) 50 min, and (c) 2 h. A clear structure evolution could be found. Basically, the shorter soaking time leads to less nanopores near the top surface due to less crosslinking and insufficient phase-separation. Increasing the soaking time leads to more nanopores besides the micron-sized ones. After 2 h soaking, the hierarchical pore structure is fully developed. (d) SEM image of the ammonia treated porous membrane (in c) after soaked in acetic acid ($C_{CH3COOH}$ - 7.5×10^{-3} M) for 0.5 h. It indicates that the morphologies of the current porous membrane have little-to-no change in actuation process.

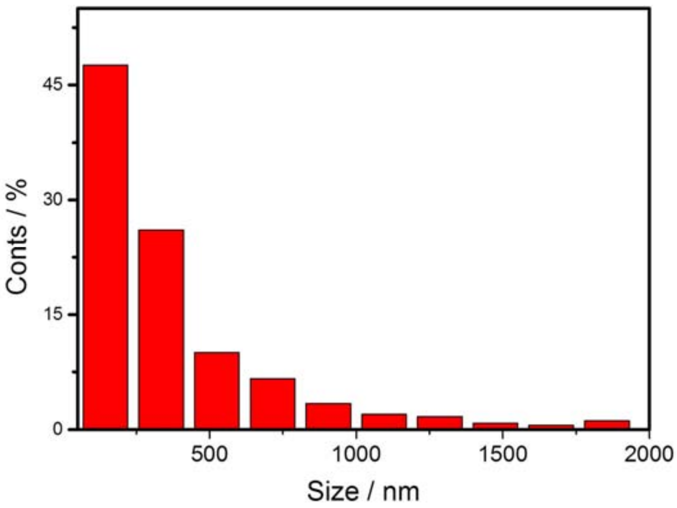

Supplementary Figure 8 | Pore size distribution of the Ptriaz-TA membrane obtained by statistic counting of the pores identified in the SEM images in Supplementary Figure 7c.

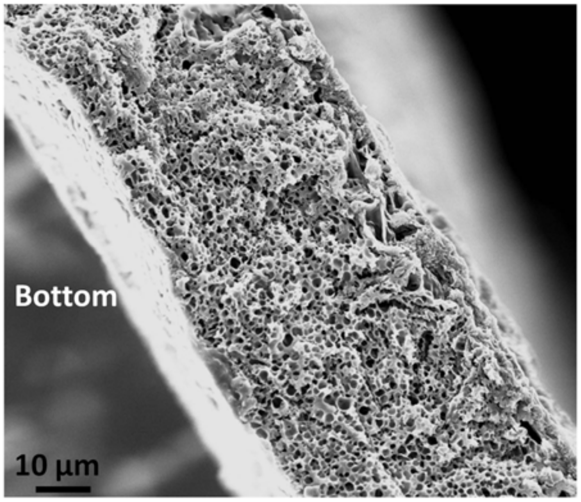

Supplementary Figure 9 | Cross-section SEM image of a porous Ptriaz-TA membrane. It was obtained from a liquid Ptriaz and TA blend film after thermo-treatment at 80 °C for 10 min (the polymer blend film was not thoroughly dried in its interior while the solvent on the surface of membrane has vanished), which was then soaked in an *a.q.* ammonia solution for 2 h. Such process was conducted to explore the influence of the drying time on the membrane pore structure. The pores in dominant micron size could be observed. Such process is perhaps due to the residual DMF left in the blend film, which interferes the phase separation process with water, making larger pores. Moreover, the actuation performance of membranes carrying such

large micron-sized pores is much poorer than that of membrane obtained by drying the polymer blend film for 2 h, which has smaller pore size.

We also test the influence of NH_3 concentration on the pore structure of membranes. The membrane structure seems to be less sensitive towards NH_3 concentration in water. The reason is, the neutralization of acids occurs through the formation of OH^-. NH_3 in aqueous solution exists as a moderate base $NH_4^+OH^-$, and the pH saturates around 11.6 at room temperature.

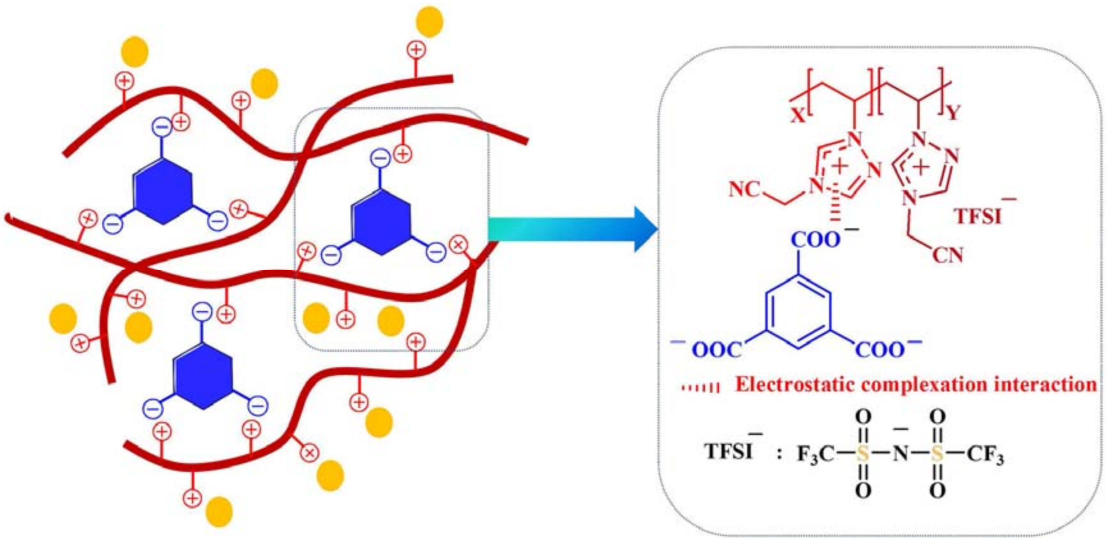

EC=X/(X+Y)………………………….(1)

EC=(415S-64)/(212S-64)……………..(2)

Supplementary Figure 10 | (top) A scheme for defining the degree of electrostatic complexation (EC) of the membrane; (bottom) equations for defining (eq. 1) and calculating (eq. 2) EC. Thus, EC is expressed in equation (1); whereas, **X** denotes the 1,2,4-triazolium units that undergo EC with $COO^-NH_4^+$ groups on TA; **Y** denotes the 1,2,4-triazolium units that are NOT involved in the EC. Note that the EC is accompanied by the release of $TFSI^-$ anions. Thus the value of EC can be calculated from the content of sulfur element because sulfur exists only in $TFSI^-$ counter anion. As such EC values at different locations of the membrane are experimentally determined by equation (2), in which **S** is the sulfur content at different locations of the membrane cross-section measured by EDX. Supplementary Figure 9 shows the sulfur content along the membrane cross-section; indicating that the EC decreases with the top-down depth along the cross-section. This EC gradient is consistent with the membrane formation

mechanism. Ammonia diffuses into the membrane from the top surface (membrane-liquid interface) and deprotonates the COOH groups on TA into carboxylate groups ($COO^{-}NH_4^{+}$), thus the EC is higher at the places closer to the top surfaces.

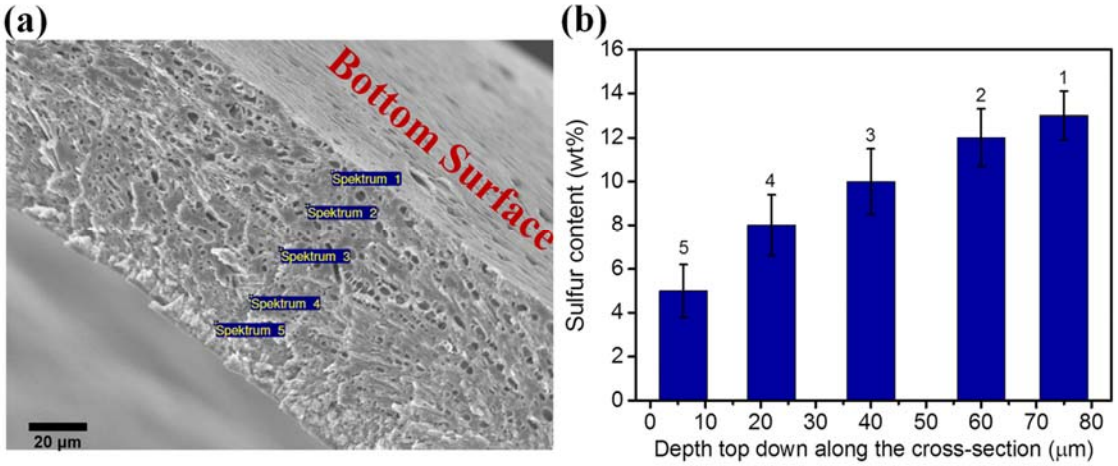

Supplementary Figure 11 | EDX analysis of cross-section of the membrane. (a) Sulfur element content at different locations along the cross-section of the membrane actuator from the top to the bottom. (b) A histogram scheme of measured sulfur content by energy dispersive x-ray spectroscopy (EDX) (The method for statistical analysis is based on "arithmetic mean", which is also used to other data analysis in the manuscript).

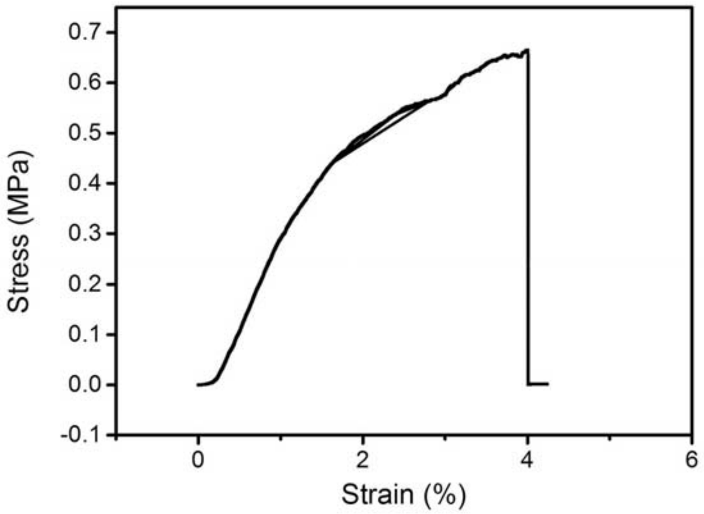

Supplementary Figure 12 | Tensile testing experiment showing the stress-strain curve (membrane stripe dimension 1mm × 25 mm × 50 μm).

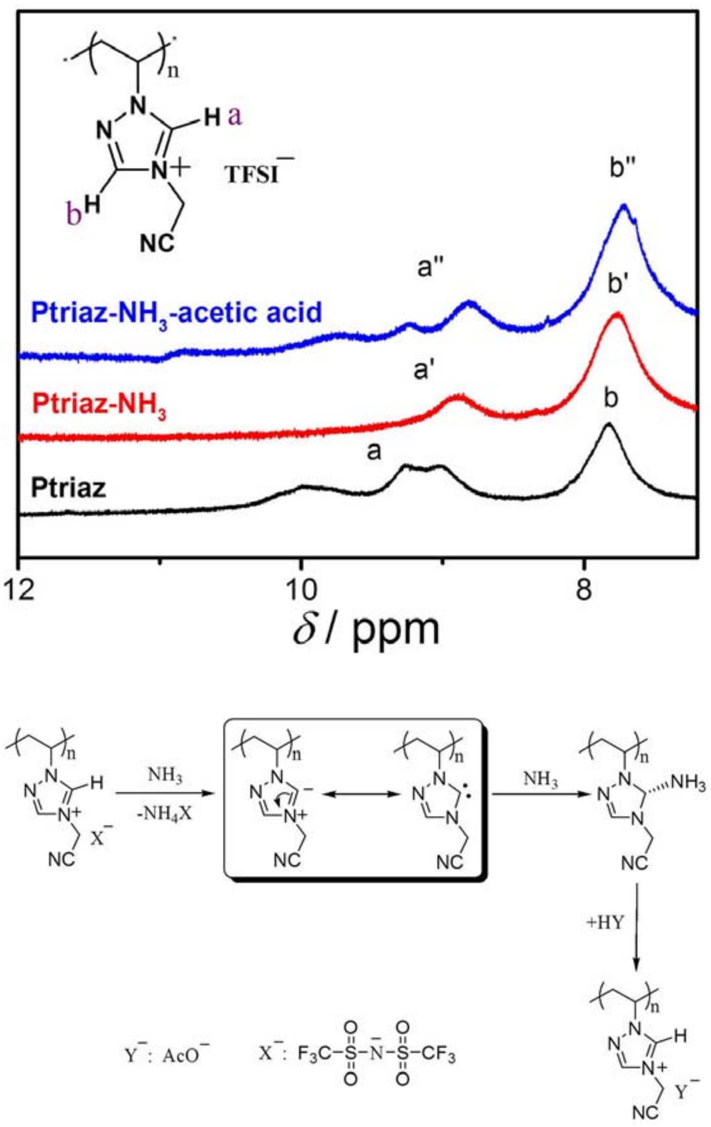

Supplementary Figure 13 | ^{1}H NMR spectra recording the reversible formation-decomposition of CNA in Ptriaz. Top part: The ^{1}H NMR spectra change of Ptriaz in *d_6*-DMSO upon consecutively adding *a.q.* NH_3 and acetic acid (note: the *a.q.* NH_3 or acetic acid treated samples were first purified to remove NH_4Cl and then dried before dissolving in *d_6*-DMSO for ^{1}H NMR measurement). Bottom part: illustrating the proposed structural variation of Ptriaz.

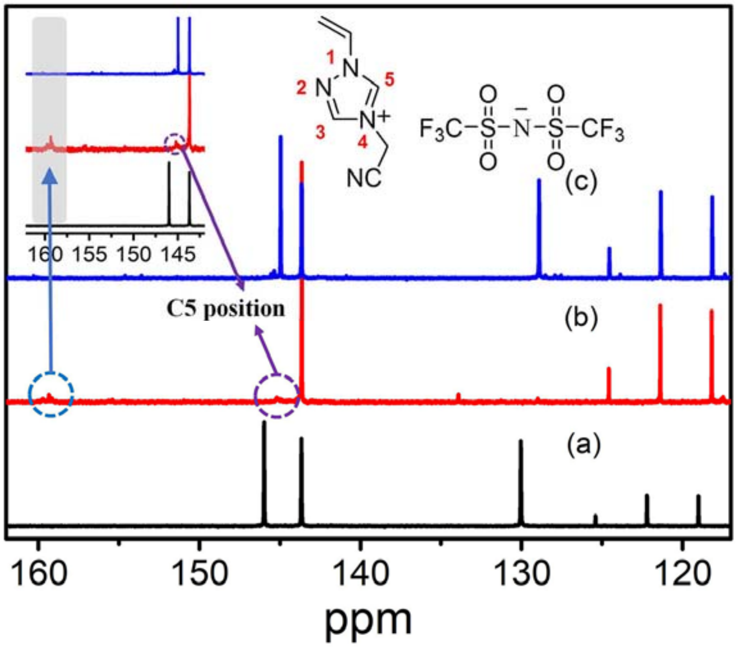

Supplementary Figure 14 | ^{13}C NMR spectra recording the reversible formation-decomposition of CNA in triazolium monomer. (a) The triazolium monomer, (b) the triazolium monomer upon adding *a.q.* NH_3 and (c) the NH_3 treated sample upon adding acetic acid (note: the Ptriaz in high concentration is not well dissolved in DMSO, so we use the triazolium monomer to detect the change of ^{13}C NMR spectra. The *a.q.* NH_3 or acetic acid treated samples were first dried before dissolving in *d*$_6$-DMSO for ^{13}C NMR measurement). Inset highlighted the spectra change of C-5 in triazolium ring by violet rectangle.

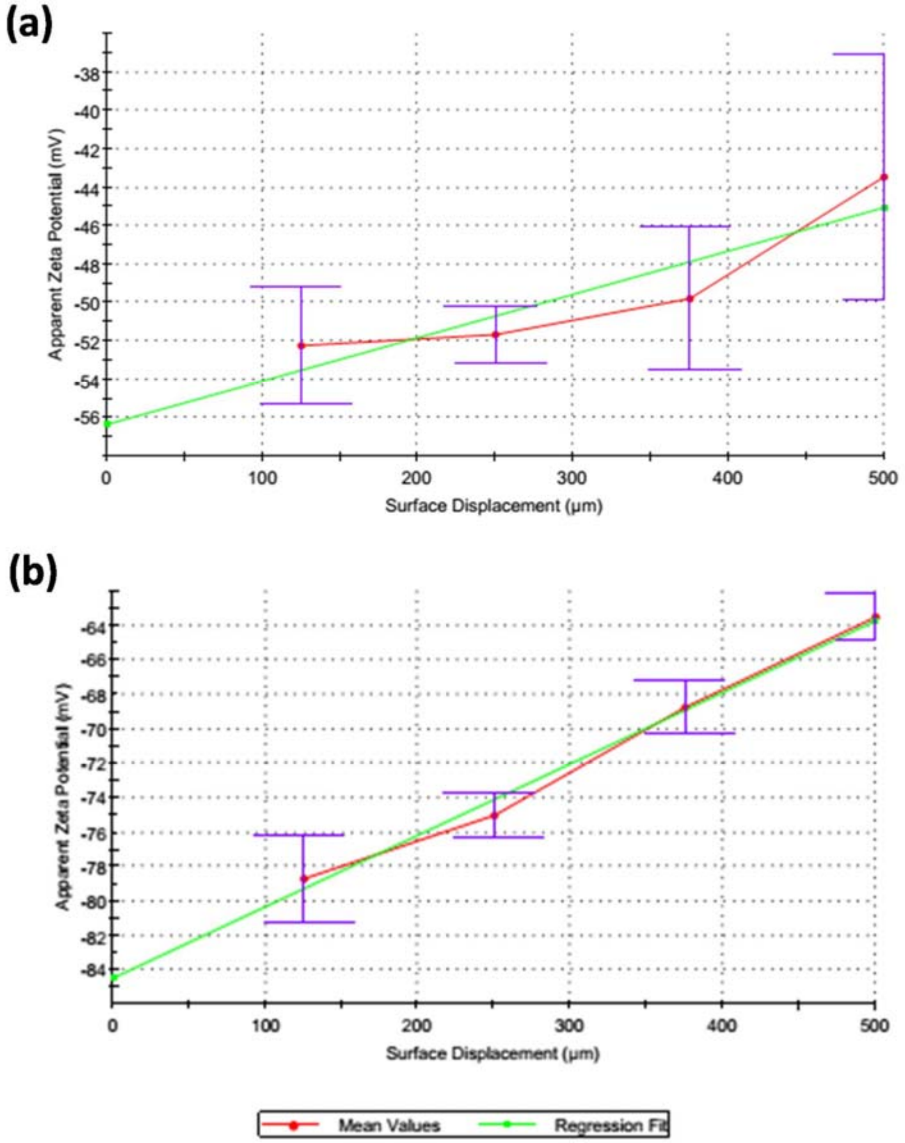

Supplementary Figure 15 | The apparent zeta potential of Ptriaz-TA membrane *vs.* displacement on (a) top and (b) bottom surfaces.

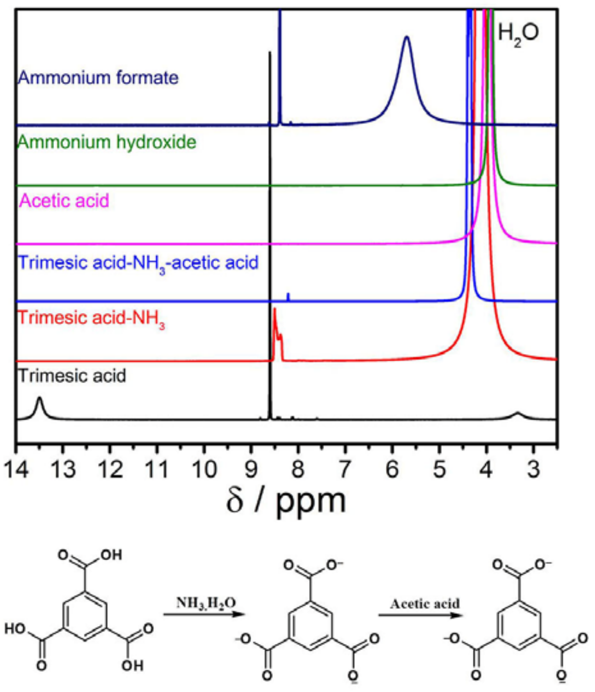

Supplementary Figure 16 | The ^{1}H NMR spectra (d_6-DMSO) recording the spectra change upon successively adding the *a.q.* NH_3 and acetic acid. The bottom part illustrated the proposed structural variation of TA**.**

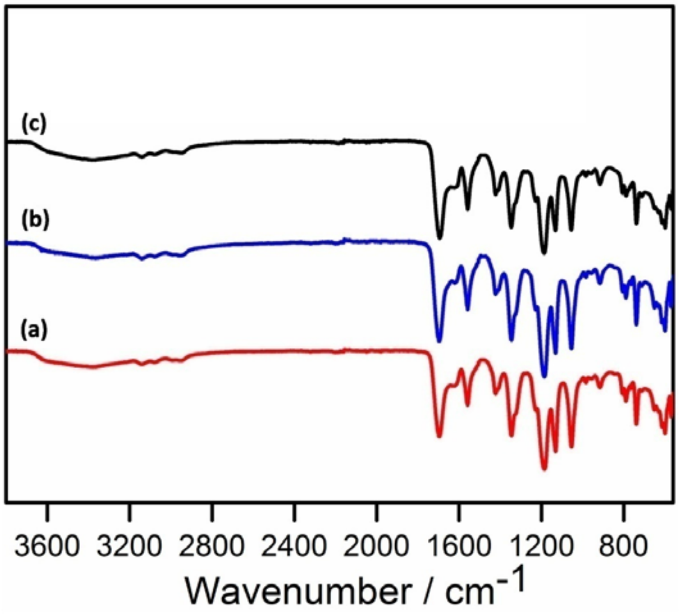

Supplementary Figure 17 | (a) The FT-IR spectra of Ptriaz-TA membrane, (b) after immersing in acetic acid, and (c) after treating (b) by *a.q.* NH_3.

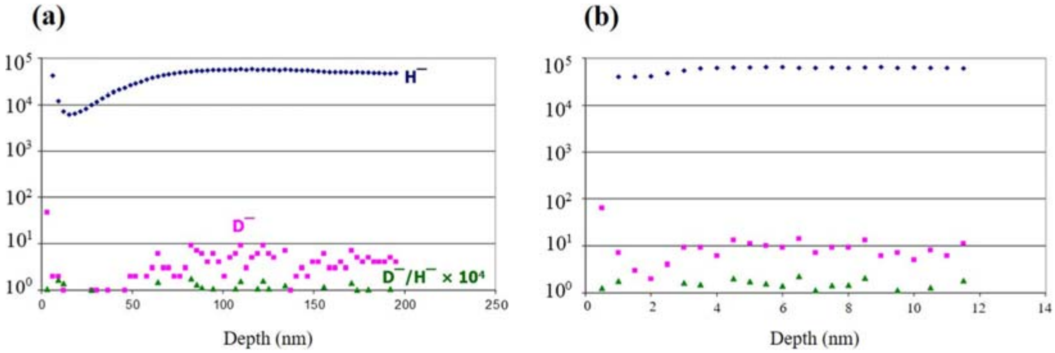

Supplementary Figure 18 | Depth profiles of H¯ and D¯ for a PMMA film (a) and an aluminum oxide film (b). This is used to check the D¯/H¯ ratios for the natural abundance of deuterium, which would have a theoretical D¯/H¯ ratio of 1.6×10^{-4}. The D¯/H¯ ratio for both materials, measured under the same conditions used to depth profile the membrane, is 0.8 and 1.4, respectively. These results for PMMA and aluminum oxide are not far away from the theoretical value, verifying that the deuterium levels detected in the membrane are due to incorporated deuterium.

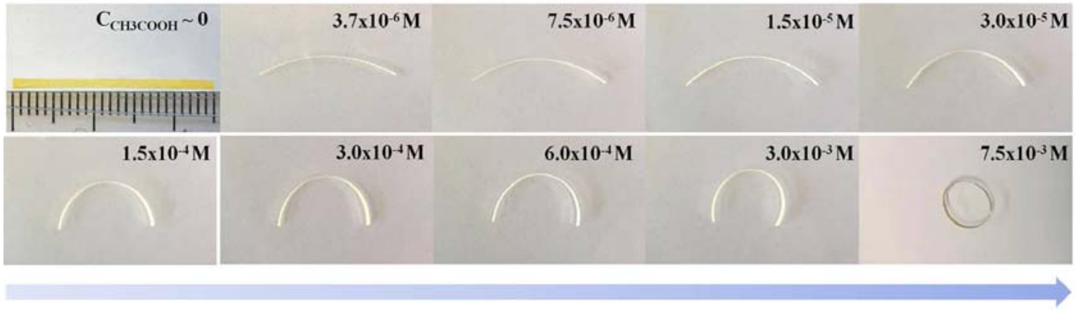

Supplementary Figure 19 | Shape deformation of Ptriaz-TA membranes (1 mm × 25 mm × 50 μm) in response to the concentration of acetic acid in aqueous solution.

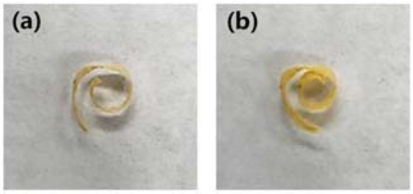

Supplementary Figure 20 | Shape deformation of Ptriaz-TA membranes (1 mm × 25 mm × 50 μm) in response to acetic acid in aqueous solution at 1.0×10^{-2} M (a) , and 1.25×10^{-2} M (b). An enhanced curvature with multicircles could be observed, while the saturating concentration is around 1.25×10^{-2} M. Therefore, the dynamic range of actuation is calculated to be ～ 3×10^{3} based on the scope of concentration for actuation.

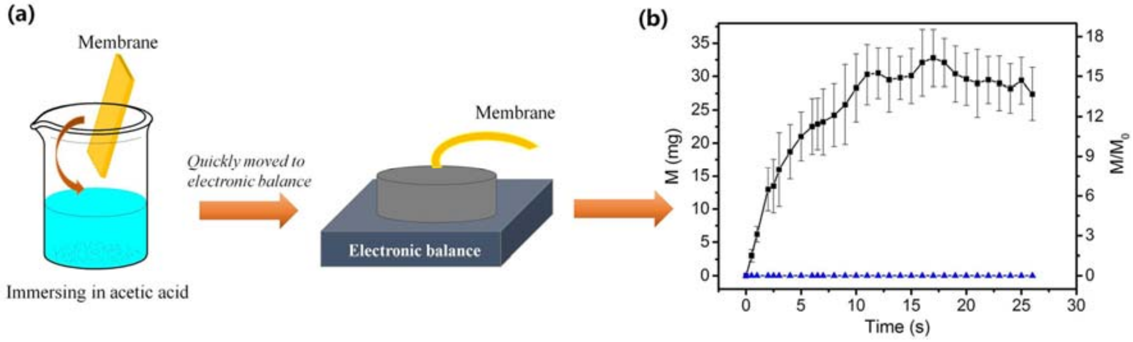

Supplementary Figure 21 | (a) Schematic illustration of the experimental force measurement set-up. (b) The plot of the force generated by membrane actuator against time. The membrane was first immersed in 7.5× 10^{-3} M *a.q.* CH_3COOH solution, then taken out with the excessive liquid being wiped away, and placed above an electronic balance. The recorded data was plotted into a black curve. M is the force that the actuator exerted on the balance; M_0 is the weight of the dried actuator membrane. Blue line: control experiment without using the actuator membrane.

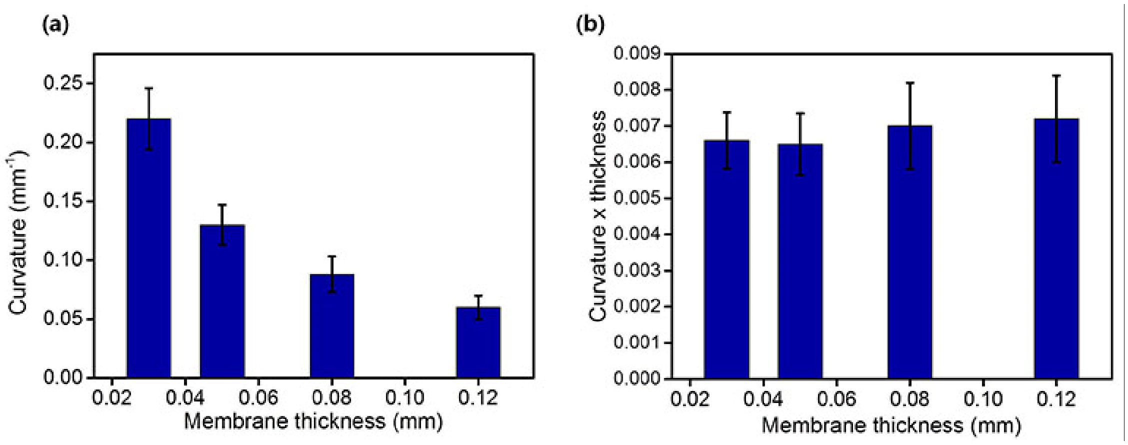

Supplementary Figure 22 | Effect of thickness of the Ptriaz-TA membrane actuator (1 mm × 25 mm) on its bending curvature in an aqueous acetic acid solution at $C_{CH3COOH}$ - 3× 10^{-4} M at room temperature (a)**.** (b) The normalized curvature (curvature multiplied by thickness).

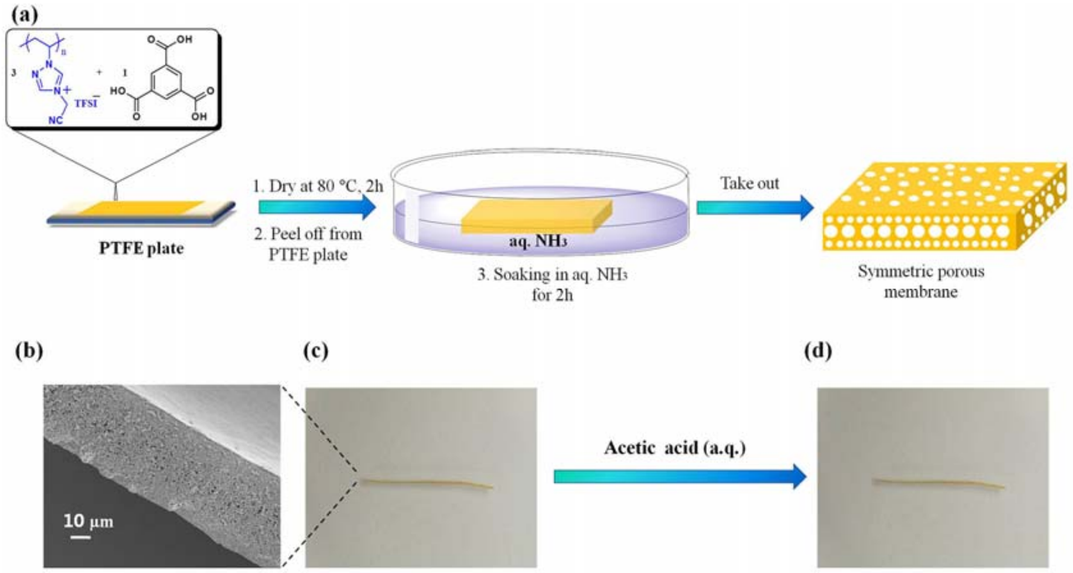

Supplementary Figure 23 | The use of symmetric Ptriaz-TA membrane to response to acetic acid. (a) The procedure for the preparation of symmetric Ptriaz-TA membrane (PTFE= Polytetrafluoroethylene). (b) The SEM image of the Ptriaz-TA. The as-synthesized symmetric membrane before (c) and after (d) immersing in an aqueous acetic acid solution ($C_{CH3COOH}$ - 7.5 × 10^{-3} M).

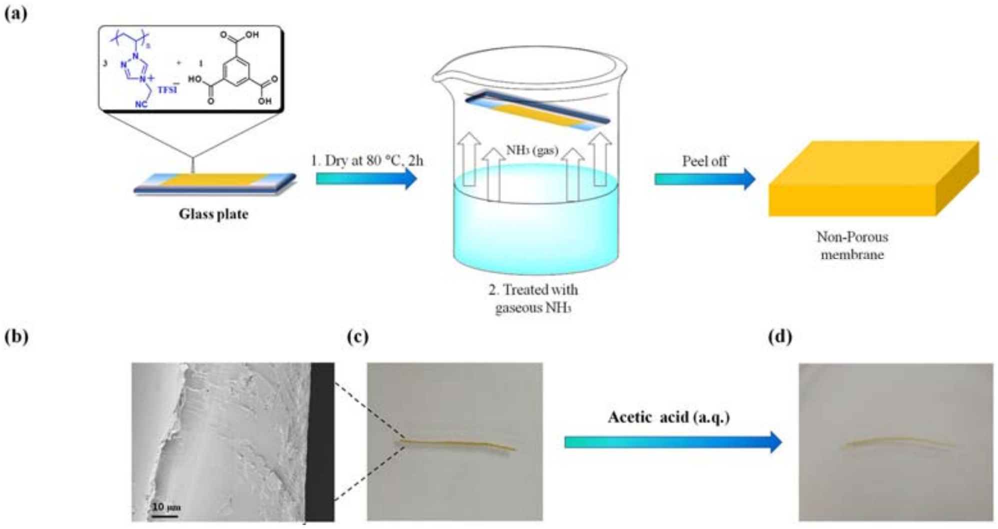

Supplementary Figure 24 | The use of non-porous Ptriaz-TA membrane to respond to acetic acid. (a) The procedure to prepare the non-porous Ptriaz-TA membrane. (b) The SEM image of cross-section of membrane. The as-sythesized membrane before (c) and after (d) immersing in an aqueous acetic acid solution at $C_{CH3COOH}$ -7.5 × 10^{-3} M for 8s.

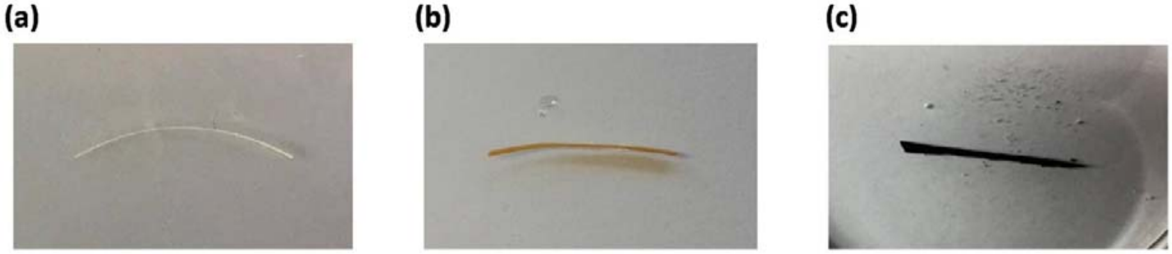

Supplementary Figure 25 | Photos of the Ptriaz-TA (a), PIm-TA (b) and PPy-TA (c) membrane (all in same size: 1mm × 25 mm × 50 μm) upon soaking in an aqueous acetic acid solution at $C_{CH3COOH}$–3.7×10^{-6} M.

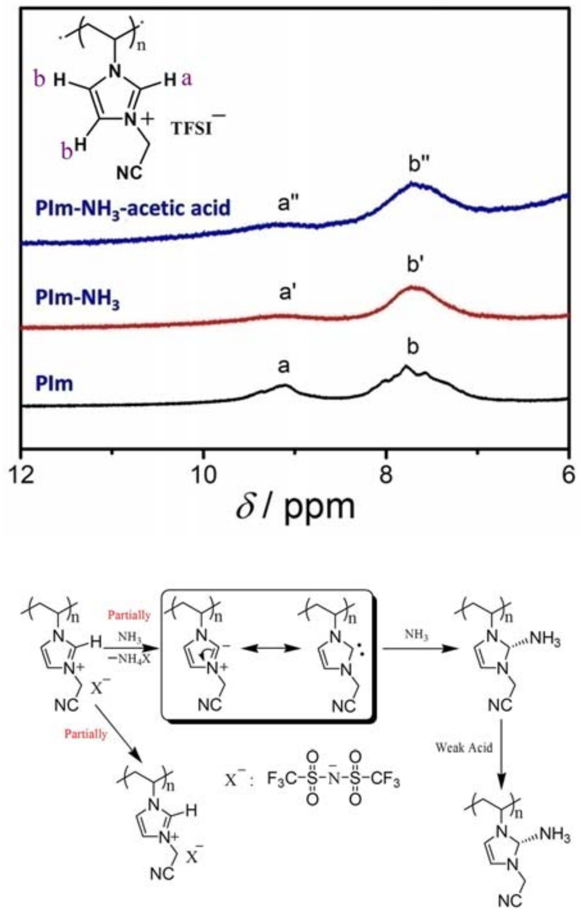

Supplementary Figure 26 | ^{1}H NMR spectra recording the formation-decomposition of CNA in PIm. Top part: The ^{1}H NMR spectra change of PIm in d_6-DMSO upon consecutively adding *a.q.* NH_3 and acetic acid (note: the *a.q.* NH_3 or acetic acid treated samples were first purified to remove NH_4Cl and then dried before dissolving in d_6-DMSO for ^{1}H NMR measurement). Bottom part: illustrating the proposed structural variation of PIm.

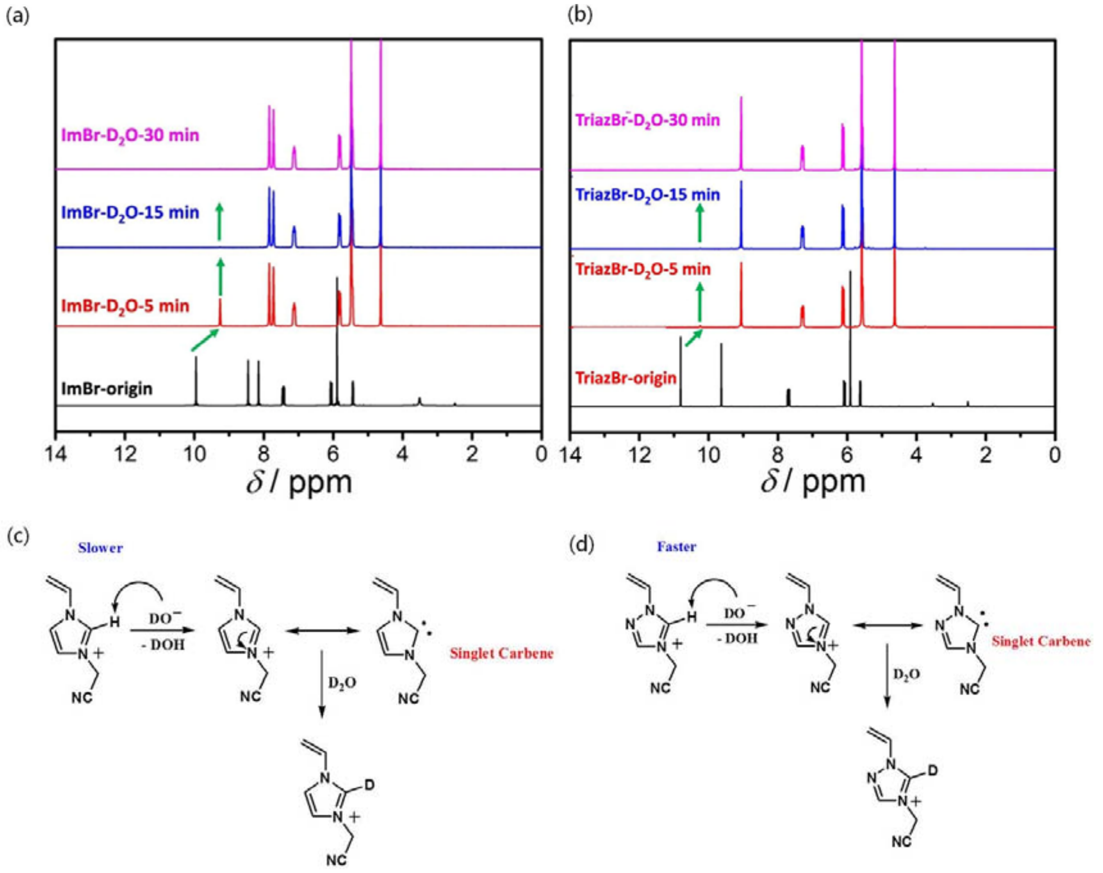

Supplementary Figure 27 | Time-dependent ^{1}H NMR spectroscopy test of isotope exchange at (a) C-2 of imidazolium and (b) C-5 of 1,2,4-triazolium cations. The experiment was conducted by dissolving 100 mg of monomer in 0.5 ml D_2O. The bottom part illustrated the proposed deuterium exchange mechanism of (c) imidazolium unit and (d) 1,2,4-triazolium unit.

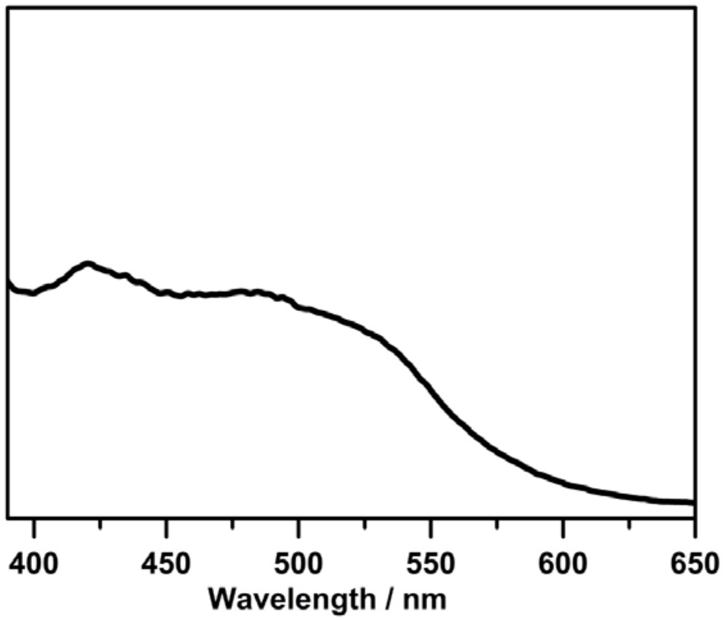

Supplementary Figure 28 | The luminescence spectrum of Ptriaz polymer by treatment with *a.q.* NH_3 solution (λ_{ex} = 365 nm).

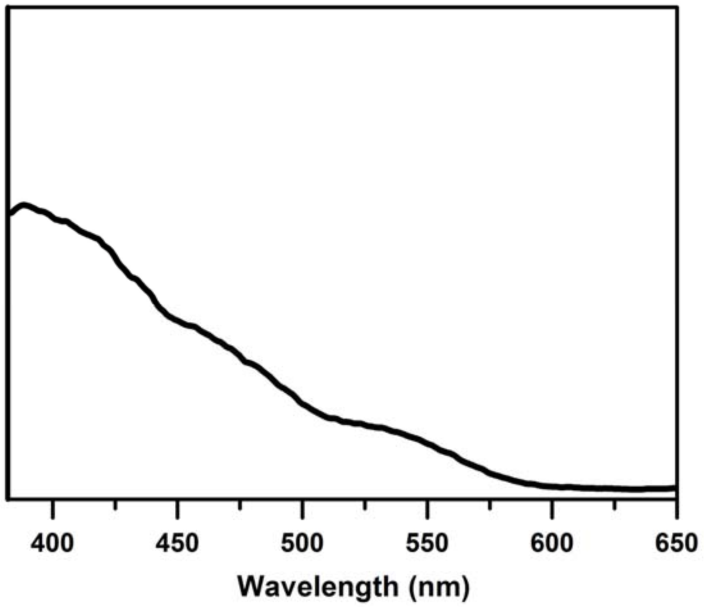

Supplementary Figure 29 | The luminescence spectrum recorded from the sample by immersing the *a.q.* NH_3 treated Ptriaz polymer (used in Supplementary Figure 28) in *a.q.* CH_3COOH solution (λ_{ex} = 365 nm). The change of the emission spectra caused by protonation-deprotonation could be attributed to variation in the electronic struture, which has been observed in previous literatures[1, 2].

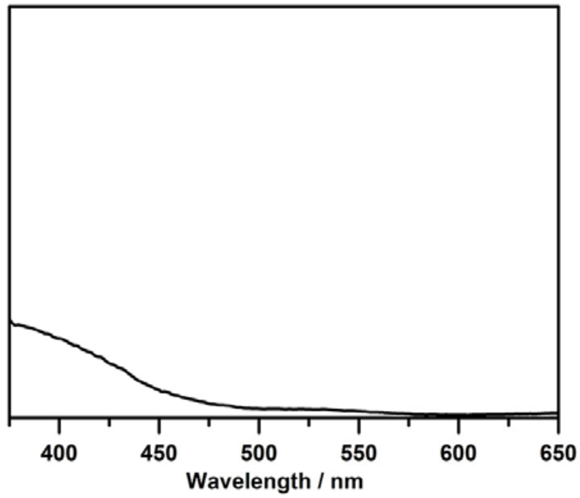

Supplementary Figure 30 | The luminescence spectrum of TA in water (λ_{ex} = 300 nm). It is shown that the emission peak located below 350 nm. Moreover, the amount of TA in the as-synthesized membrane is less than 5 wt% as demonstrated in our previous work[3]. Since the protonation by CH_3COOH will not occur on TA, the actuation-introduced emission change by TA is negligible.

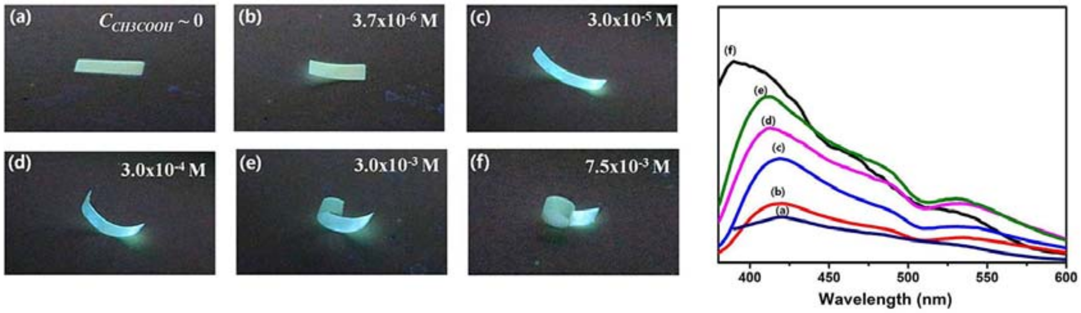

Supplementary Figure 31 | Acetic acid concentration-dependent emission spectra (image on the right) of Ptriaz-TA membrane stripes (images on the left) at λ_{ex} = 365 nm. The membrane stripes were immersed in *a.q.* CH_3COOH solutions at different concentration (a-f). The membranes were curled gradually forward as concentration increased from a to f. Simultaneously, the fluorescent intensity increased and was accompanied with a blue shift of the emission peak, which can be explained that at a higher concentration of *a.q.* CH_3COOH solution more CNA units in the membrane were transferred into 1,2,4-triazolium. Finally, the luminescence profile of CH_3COOH-treated membrane (f) is consistent with that of native Ptriaz polymer treated by first *a.q.* NH_3 and then *a.q.* CH_3COOH solution (Supplementary Figure 29).

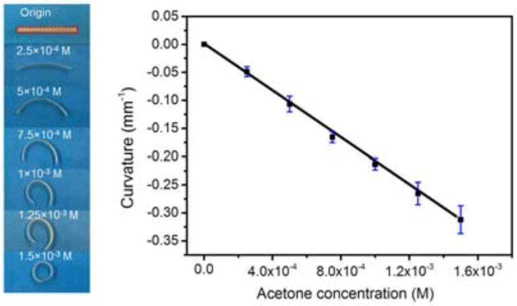

Supplementary Figure 32 | Ptriaz-TA membrane acutator responses to acetone. Left column: shape defromation of a Ptriaz-TA membrane (1mm × 25 mm × 120 μm) in response to the molar amount of the acetone in aqeous solution (Note: the membrane on the up was a top view and the rest was side view. The red dotted line indicates the membrane top surface). Right: plot of curvature (mm^{-1}) of the membrane actuator against acetone concentration. Please note: the picture for each membrane was taken in 30 s after quickly mixing acetone with water solution in different concentration. The negative value of curvature means the membrane bending with top surface inwards.

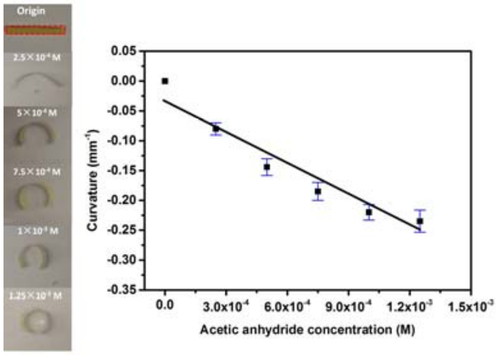

Supplementary Figure 33 | Ptriaz-TA membrane acutator responses to acetic anhydride. Left column: shape defromation of a Ptriaz-TA membrane (1mm × 25 mm × 120 μm) in response to the molar amount of the acetic anhydride in aqeous solution (Note: the membrane on the up was a top view and the rest was side view. The red dotted line indicates the membrane top surface). Right: plot of curvature (mm^{-1}) of the membrane actuator against acetic anhydride concentration. Please note: to aviod the influence of the hydrolysis process, the picture for each membrane was taken within 3 s after quickly mixing acetic anhydride with water solution in different concentration. The negative value of curvature means the membrane bending with top surface inwards.

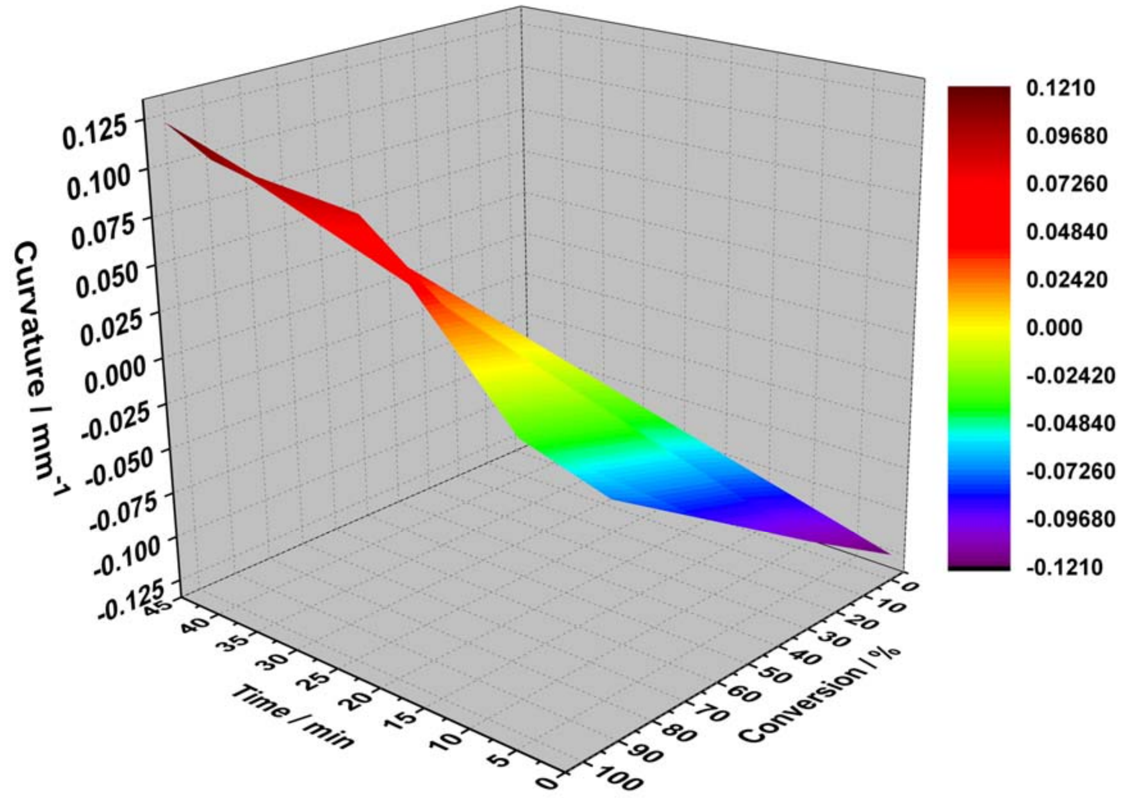

Supplementary Figure 34 | Time-conversion-curvature related calibration surface for monitoring the entire reaction of hydrolysis of acetic anhydride in water.

Supplementary Table 1 | Comparison of the sensitivity of Ptriaz-TA actuator with relevant literature data.

Materials	Proton concentration (M)	Materials thickness (mm)	Curvature (mm^{-1})	Apparent sensitivity [a]	Normalized sensitivity [b]	Time (min)	Ref
Flash-welded asymmetric film of dedoped polyaniline nanofibers	0.5	0.03	0.25	0. 5	0.015	0.33	[4]
PDMA-co-PAPMA-brush-grafted pPVC substrate film	1	0.5	0.26	0.26	0.13	--	[5]
PEG-30%-MDI-DMPA film	0.05	0.1	0.08	1.6	0.16	60	[6]
Hydrogel laminate gel	10^{-2}	1.5	0.11	11	16.5	30	[7]
PEG–MDI–BIN film	10^{-2}	0.5	0.12	12	6	120	[8]
Bilayered hydrogel	10^{-3}	1	0.055	55	55	140	[9]
Bio-hydrogel	0.1	6	0.11	1.1	6.6	2.67	[10]
ACAT–vitrimer	0.1	0.2	0.05	0.5	0.1	4	[11]
3D hydrogels	10^{-2}	0.5	0.35	35	17.5	10	[12]
**Ptriaz-TA membrane**	**3.7×10^{-6}**	**0.05**	**0.042**	**11351**	**567**	**0.5**	**This work**

a: Apparent sensitivity is defined as the change of curvature achieved by adding 1M proton containing solution, i.e., the curvature (C) divided by the proton concentration (P): C/P. Please note: The proton responsive materials gave here for sensitivity comparison means the material is straight in water and bends upon immersing in proton containing solution. The comparison of the curvature of actuation occurred at room temperature.

b: Normalized sensitivity equals apparent sensitivity multiplied by thickness.

PDMA-co-PAPMA: poly(N,N-dimethylacrylamide-co-aminopropyl-methacrylamide)

Ppvc: poly(vinyl chloride)

PEG: poly(ethylene glycol)

MDI: diphenylmethanediisocyanate

BIN: N,N-bis(2-hydroxylethyl) isonicotinamine

ACTA: aniline trimer

Supplementary Table 2 | Relations of pK_a values and curvature of membrane for each weak acid.

Acids	pK_a	Curvature (mm^{-1})
Citric Acid	3.15	0.2792±0.01
Formic Acid	3.74	0.2443±0.015
L-Aspartic Acid	3.90	0.2059±0.012
3,3'-Dithiodipropionic Acid	4.03	0.185±0.013
L-Ascorbic Acid	4.17	0.1759±0.014
3-Mercaptopropionic Acid	4.34	0.1466±0.012
6-Aminohexanoic Acid	4.43	0.1256±0.009
Methacrylic Acid	4.65	0.1117±0.011
Acetic Acid	4.76	0.08866±0.01
Pivalic Acid	5.02	0.07679±0.011

Supplementary Table 3 | The time dependent relative content of acetic anhydride in the reaction of hydrolysis of acetic anhydride as well as temporally recorded curvature of the membrane are summarized (Note: positive value of curvature means the membrane is bending with the bottom part inward, and *vice versa*).

Time (min)	Acetic anhydride (mol%)	Curvature (mm^{-1})
0	100	-0.121±0.013
5	87.0	-0.110±0.012
10	63.9	-0.085±0.009
15	50.9	-0.073±0.008
20	36.3	-0.036±0.005
25	29.4	0.03±0.004
30	17.5	0.081±0.009
35	10	0.088±0.01
40	1.2	0.107±0.011
45	0	0.121±0.01

Supplementary Methods

Synthesis of poly(ionic liquid)s

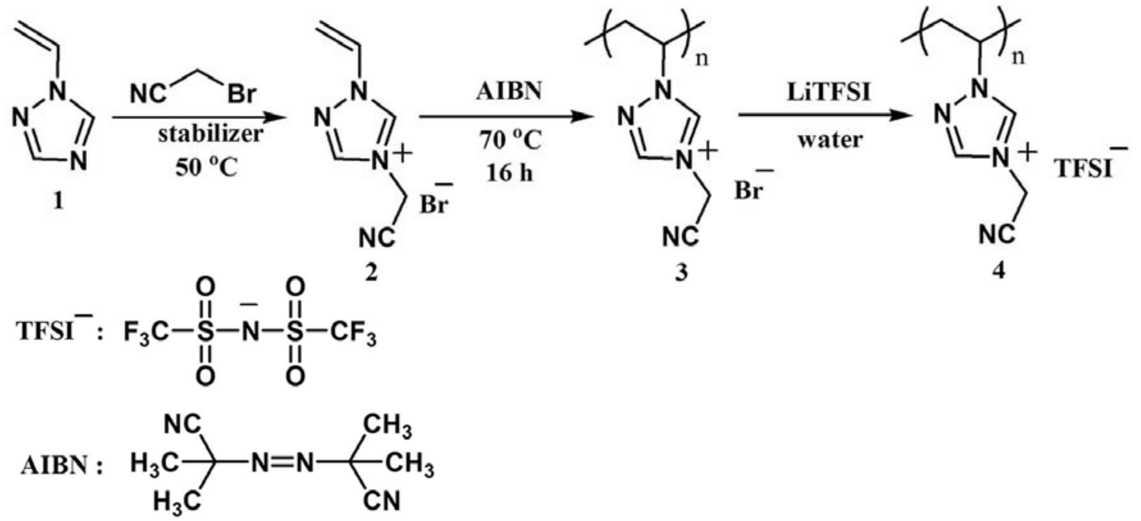

Synthetic route towards poly(4-cyanomethyl-1-vinyl-1,2,4-triazolium bis(trifluoromethanesulfonyl)imide) (Ptriaz).

Synthesis of monomer 2: A mixture of 1-vinyl-1,2,4-triazole **1** (5 mL, 5.5g, 57.83 mmol) and a 1.5 equivalent amount of bromoacetonitrile was added into a 50 mL round flask, accompanied with 2,6-di-tert-butyl-4-methylphenol (50 mg, 0.227 mol) as the stabilizer. After heated to and kept at 55 °C for 3 days, pale white solids were obtained, which was washed by diethyl ether for three times.

Synthesis of polymer 3: A mixture of monomer **2** (15 g, 69.77 mmol) and AIBN (1.5 mol%) as initiator was added to anhydrous DMSO (concentration: ~1 g monomer in 15 mL solvent) inside a 500 mL round-bottom schlenk flask. The flask was treated with three freeze-pump-thaw cycles and finally purged with argon. The reaction was stirred at 70 °C for 16 h under argon atmosphere. Afterwards, the products were precipitated in tetrahydrofuran (THF) and washed with hot methanol overnight by Soxhlet extractor. Yellow powders were obtained after vacuum drying process.

Synthesis of polymer 4: Anion exchange was performed by dropwise addition of solution 2) into solution 1) : 1) 10 g of polymer **3** dissolved in 1000 mL of deionized water, and 2) 1.05 eq. of lithium bis(trifluoromethanesulfonyl)imide in 100 mL deionized water. White precipitate

appeared, and then it was filtered off and washed by deionized water for 3 times before it was dried in vacuum oven (80 °C).

The preparation methods of poly(3-cyanomethyl-1-vinyl-imidazolium bis(trifluoromethanesulfonyl)imide) (denoted as “PIm”) and poly(1-cyanomethyl-4-vinyl-pyridinium bis(trifluoromethanesulfonyl)imide) (denoted as “PPy”) were described in the previous literatures.[13]

4-Cyanomethyl-1-vinyl-1,2,4-triazolium bromide (2): (Yield: 95%, 11.80 g): ^{1}H NMR (400 MHz, DMSO-d_6, δ, ppm): 10.54 (s, 1H), 9.52 (s, 1H), 7.60 (dd, 1H, J_1=16 Hz, J_2=8 Hz), 6.05 (d, 1H, J=16 Hz), 5.80 (s, 2H), 5.58 (d, 1H, J=8 Hz); ^{13}C NMR (400 MHz, DMSO-d_6, δ, ppm): 159.10, 136.58, 129.50, 74.58, 32.97, 24.34.

Poly(4-cyanomethyl-1-vinyl-1,2,4-triazolium bromide) (3): (Yield: 81%, 12.15 g): ^{1}H NMR (400 MHz, DMSO-d_6, δ, ppm): 10.10 (br, 1H), 9.12 (br, 1H), 7.86 (br, 2H), 4.72 (m, 1H), 2.03 (br, 2H).

Poly(4-cyanomethyl-1-vinyl-1,2,4-triazolium bis(trifluoromethanesulfonyl)imide) (4): (Yield: 98%, 19.01 g): ^{1}H NMR (400 MHz, DMSO-d_6, δ, ppm): 9.95 (br, 1H), 9.10 (br, 1H), 7.82 (br, 2H), 4.72 (m, 1H), 1.98 (br, 2H).

Calculation of pKa of Ptriaz. Acid dissociation constant (pK_a value) of Ptriaz was measured by Sirius Analytical Ltd., Britain (http://www.sirius-analytical.com/). The sample pK_a was investigated using the spectrometric (Fast-UV) and potentiometric (pH-metric) techniques.

Yasuda-Shedlovsky result

Extrapolation type	pKa 0%	SD	Intercept	Slope	R^2	Ionic strength	Temperature
Yasuda-Shedlovsky	8.62	±0.04	-19.32	2333.3982	0.9987	0.197 M	24.8℃

Graphs

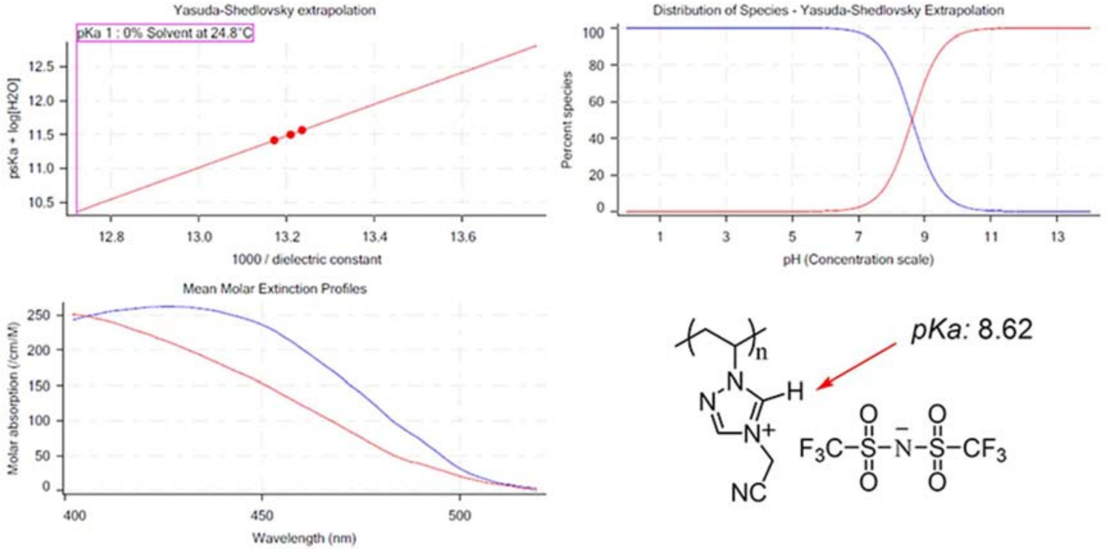

Illustrating the membrane curvature calculation. L is the length of the actuator; θ and r are the central angle and radius of the bended arch, respectively. The r and curvature can be calculated by equation: $r = (180L/\theta\pi)$; Curvature = 1/r.

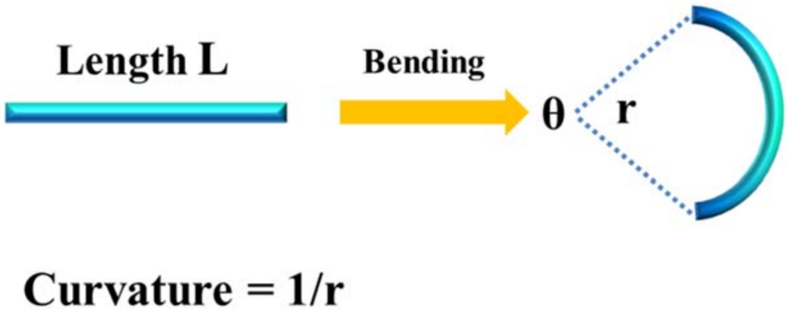

Supplementary References

1. Berezin, M. Y., Kao, J. & Achilefu, S. pH-dependent optical properties of synthetic fluorescent imidazoles. *Chem. Eur. J.* **15**, 3560–3566 (2009).
2. Wang, B.Y., Chen, Z. Q., Huang, H. & Cao, Q. Y. Fluorescent triazolium for sensing fluoride anions in semi-aqueous solution. *RSC Adv.*, **7**, 43950–43956 (2017).
3. Sun, J.K. *et al*. A tale of two membranes: from poly (ionic liquid) to metal–organic framework hybrid nanoporous membranes via pseudomorphic replacement. *Mater. Horiz.* **4**, 681–687 (2017).
4. Baker, C. O. B. *et al.* Monolithic actuators from flash-welded polyaniline nanofibers. *Adv. Mater.* **20**, 155–158 (2008).
5. Zou, Y. *et al.* Bending and stretching actuation of soft materials through surface-initiated polymerization. *Angew. Chem. Int. Ed.* **50**, 5116–5119 (2011).
6. Chen, H. *et al.* Highly pH-sensitive polyurethane exhibiting shape memory and drug release. *Polym. Chem.* **5**, 5168–5174 (2014).
7. Ma, C. *et al.* Supramolecular lego assembly towards three-dimensional multi-responsive hydrogels. *Adv. Mater.* **26**, 5665–5669 (2014).
8. Song, Q. *et al.* Thermo- and pH-sensitive shape memory polyurethane containing carboxyl groups. *Polym. Chem.* **7**, 1739–1746 (2016).
9. Hu, Y. *et al.* Reversible modulation of DNA-based hydrogel shapes by internal stress interactions. *J. Am. Chem. Soc.* **138**, 16112–16119 (2016).
10. Duan, J. *et al.* Bilayer hydrogel actuators with tight interfacial adhesion fully constructed from natural polysaccharides. *Soft Matter*, **13**, 345–354 (2017).
11. Chen, Q. *et al.* Multi-stimuli responsive and multi-functional oligoaniline-modified vitrimers. *Chem. Sci.* **8**, 724–733 (2017).
12. Ma, C. *et al.* A multiresponsive anisotropic hydrogel with macroscopic 3D complex deformations. *Adv. Funct. Mater.* **26**, 8670–8676 (2016).
13. Tauber, K., Lepenies, B. & Yuan, J. Polyvinylpyridinium-type gradient porous membranes: synthesis, actuation and intrinsic cell growth inhibition. *Polym. Chem.* **6**, 4855–4858 (2015)
