# Supplementary material for: Porous polycarbene-bearing membrane actuator for ultrasensitive weak-acid detection and real-time chemical reaction monitoring
Source: Nat Commun. 2018 Apr 30;9:1717. doi: 10.1038/s41467-018-03938-x (PMC5928224; doi:10.1038/s41467-018-03938-x)
Supplement: Supplementary file 2 — Description of Additional Supplementary Files [file 41467_2018_3938_MOESM2_ESM.pdf]

Description of Additional Supplementary Files

File Name: Supplementary Movie 1
Description: The bending of membrane toward a.q. CH_3COOH solution.

File Name: Supplementary Movie 2
Description: The unbending of membrane toward a.q. NH_3 solution.
